# Llgl1 mediates timely epicardial emergence and establishment of an apical laminin sheath around the trabeculating cardiac ventricle

**DOI:** 10.1242/dev.202482

**Published:** 2024-06-28

**Authors:** Eric J. G. Pollitt, Juliana Sánchez-Posada, Corinna M. Snashall, Christopher J. Derrick, Emily S. Noël

**Affiliations:** School of Biosciences and Bateson Centre, University of Sheffield, Western Bank, Sheffield S10 2TN, UK

**Keywords:** Epicardium, Extracellular matrix, Heart development, Laminin, Polarity, Zebrafish

## Abstract

During heart development, the embryonic ventricle becomes enveloped by the epicardium, which adheres to the outer apical surface of the heart. This is concomitant with onset of ventricular trabeculation, where a subset of cardiomyocytes lose apicobasal polarity and delaminate basally from the ventricular wall. Llgl1 regulates the formation of apical cell junctions and apicobasal polarity, and we investigated its role in ventricular wall maturation. We found that *llgl1* mutant zebrafish embryos exhibit aberrant apical extrusion of ventricular cardiomyocytes. While investigating apical cardiomyocyte extrusion, we identified a basal-to-apical shift in laminin deposition from the internal to the external ventricular wall. We find that epicardial cells express several laminin subunits as they adhere to the ventricle, and that the epicardium is required for laminin deposition on the ventricular surface. In *llgl1* mutants, timely establishment of the epicardial layer is disrupted due to delayed emergence of epicardial cells, resulting in delayed apical deposition of laminin on the ventricular surface. Together, our analyses reveal an unexpected role for Llgl1 in correct timing of epicardial development, supporting integrity of the ventricular myocardial wall.

## Teaser

**Summary:** The polarity protein Llgl1 promotes timely deposition of laminin on the apical surface of the developing cardiac ventricle through regulating timing of epicardial colonisation in zebrafish.

## INTRODUCTION

During cardiac development the ventricular wall is initially two cell layers thick, comprising an outer layer of cardiomyocytes (CMs) and an inner layer of endocardium. As the ventricular wall matures trabeculation is initiated, a process which builds muscle mass and improves pumping efficiency in the heart. Onset of trabeculation occurs at ∼55-60 h post fertilisation (hpf) in zebrafish, the same stage as initiation of epicardium development, a mesothelial layer which envelops the outer ventricular surface and contributes multiple cell types to the mature heart (Cao et al., 2020; Peralta et al., 2014). Epicardial development is initiated by formation of proepicardial clusters at the venous pole and atrioventricular canal through delamination of cells in the dorsal pericardium. Epicardial cells are released from the proepicardial organ, move through the pericardial cavity and attach to the myocardium (Peralta et al., 2013). The concurrent timing of epicardial establishment with trabeculation suggests that there may be links between these processes. Although previous studies have demonstrated that loss of epicardium does not affect early trabeculation, zebrafish with mutations in genes required for epicardial development do exhibit defects in ventricular wall integrity, characterised by aberrant apical extrusion of cardiomyocytes into the pericardial space (Boezio et al., 2023).

The ventricular myocardial wall is an epithelium with apicobasal polarity. The basal surface (adjacent to the endocardial layer and ventricular lumen) is interior, from which cardiomyocytes will delaminate to form trabecular seeds (Gunawan et al., 2021; Staudt et al., 2014; Jiménez-Amilburu et al., 2016). The apical CM surface is the external, abluminal ventricular wall, facing the pericardial cavity (to which the epicardium will adhere). Establishment and maintenance of apicobasal polarity in epithelial cells is regulated by interactions between three complexes: the Crumbs, Scribble and Par complexes (Martin et al., 2021). The onset of trabeculation is preceded by the relocalisation of the apical protein Crumbs 2a (Crb2a) from apical CM junctions to the apical surface of CMs, suggesting trabeculation is accompanied by destabilisation of apical cell-cell junctions, which facilitates basal delamination (Jiménez-Amilburu and Stainier, 2019). *crb2a* mutant embryos display aberrantly multilayered ventricular wall CMs and trabeculation fails, demonstrating that apicobasal polarity is important in ventricular wall maturation. Apicobasal polarity of epithelia is supported by the basal deposition of the extracellular matrix (ECM) component laminin (Matlin et al., 2017; Buckley and St Johnston, 2022), and therefore a laminin-rich basement membrane may be present on the luminal ventricular surface before trabeculation, although this has not directly been shown. Basal delamination of cells in different biological contexts (e.g. cancer) is accompanied by degradation of basal laminin (Akhavan et al., 2012; Banerjee et al., 2022), suggesting that if laminin is basally deposited in the early ventricle, this may need to be (locally) degraded to support trabecular seeding. Consequently, key questions remain around whether cardiomyocyte delamination requires remodelling of basal laminin, how cell delamination remains directional and whether regulation of apicobasal polarity links these phenomena.

In *Drosophila*, Lgl [Lethal giant larvae; also known as L(2)gl] forms part of the basolateral Scribble complex and regulates timely redistribution of the apical Crumbs complex in epithelia during larval development (Martin et al., 2021; Bonello and Peifer, 2019), suggesting that vertebrate homologues of *Lgl* may also be important for apicobasal polarity in epithelia such as the ventricular wall. Zebrafish have two *Lgl* homologues, *llgl1* and *llgl2*, and *llgl1* has previously been shown to be required for early stages of heart morphogenesis (Flinn et al., 2020). Although *Llgl1* expression has also been reported in the developing mouse heart and both adult mouse and human hearts (Uhlén et al., 2015; Klezovitch et al., 2004), whether *llgl1* plays a role in ventricular wall development has not been examined.

In this study, we describe requirements for *llgl1* in maintaining ventricular wall integrity at the onset of trabeculation, and in facilitating timely establishment of the epicardial layer around the ventricle. We reveal that the epicardium deposits a layer of laminin at the apical CM surface of the ventricle and provides evidence for a similar requirement for laminin in epicardial development and maintenance of ventricular wall integrity. Together, our results reveal novel roles for apicobasal regulators in epicardial development and ventricular wall maturation during heart development.

## RESULTS

### Llgl1 regulates ventricular wall integrity and trabeculation

Establishment, maintenance and regulation of apicobasal polarity is important for ventricular wall maturation (Gunawan et al., 2021). Llgl1 is a conserved mediator of apicobasal polarity and we hypothesised it may play a role in ventricular wall maturation. We generated a novel *llgl1* mutant using CRISPR-Cas9-mediated genome editing, recovering a mutant with a 32 bp deletion in exon 2, resulting in a truncated protein lacking all Llgl1 functional domains (Fig. S1A,B). qPCR analysis revealed no change in *llgl1* mRNA levels in *llgl1* mutants compared with wild type (Fig. S1C), demonstrating that the mutant transcript was not degraded (El-Brolosy et al., 2019). *llgl1* mutants exhibit mild cardiac oedema at 72 hpf, which resolves in most embryos by 5 days post fertilisation (dpf) (Fig. S1D-I). mRNA *in situ* hybridisation analysis of heart looping revealed a variable reduction in looping morphogenesis in *llgl1* mutants compared with wild type at 48 hpf (Fig. S2A-H), in line with previously-described heart phenotypes in *llgl1* mutants and morphants (Flinn et al., 2020). We further analysed heart morphology using live light-sheet microscopy of *Tg(myl7:LifeActGFP);Tg(fli1a:AC-TagRFP)* double transgenic embryos, allowing visualisation of myocardium (green) and endocardium (magenta). Analysis of heart morphology between 55 hpf and 120 hpf revealed that *llgl1* mutants continue to exhibit defects in heart morphogenesis (Fig. S2I-N). Although *llgl1* mutants exhibit changes in general cardiac morphology, quantification of heart size, myocardial tissue volume and lumen capacity revealed no significant changes compared with siblings (Fig. S2O-S).

Despite the fact that *llgl1* mutants have morphological heart defects, they are adult viable. We observed no overt morphological abnormalities in adult *llgl1* mutants (Fig. S3A,B), and found adult mutants at approximately Mendelian ratios in a colony grown from *llgl1* heterozygous incross embryos (wild type *n*=8, *llgl1^+/−^ n*=11, *llgl1^−/−^ n*=6). As *llgl1* mutants exhibit variable heart morphology at 55 hpf, we performed light-sheet imaging of *llgl1* mutant embryos at 120 hpf, separated larvae into mild and severe cardiac phenotypes, and raised each category to adulthood. Adults raised from *llgl1* mutants with either mild or severe larval heart phenotypes had comparable morphology and behaviour. Swim tunnel analysis of *llgl1* mutant exercise tolerance revealed no deficits compared with wild-type siblings, suggesting cardiovascular performance was not compromised (Fig. S3C), and dissected *llgl1* mutant adult hearts appeared to be grossly normal. However, *llgl1* mutant fish responded poorly to anaesthesia, including gill bleeding (*n*=3/4), delayed recovery (*n*=3/4) and death (*n*=2/4), phenotypes which were never observed in wild-type or heterozygous animals (*n*=6).

**Fig. 1.**
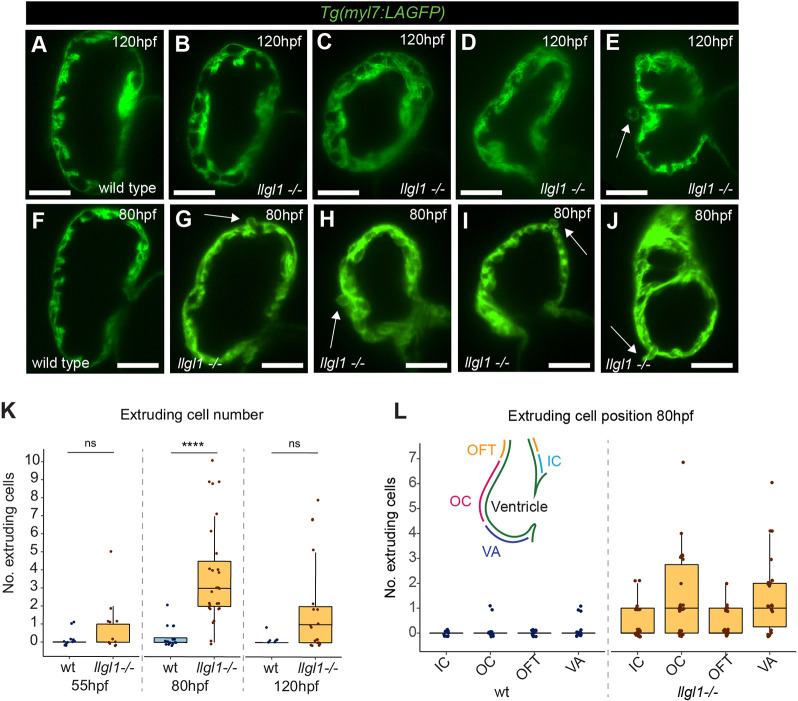
**Llgl1 promotes organised trabeculation and ventricular wall integrity.** (A-J) Live light-sheet *z*-slices through the ventricle of *Tg(myl7:LifeAct-GFP)* transgenic wild-type and *llgl1* mutant embryos visualising the myocardium at 120 hpf (A-E) and 80 hpf (F-J). Scale bars: 50 μm. Wild-type embryos have regular trabeculae emerging predominantly from the outer curvature of the ventricular wall at 120 hpf (A), whereas *llgl1* mutant embryos exhibit disorganised trabeculae, ranging from wild-type-like (B), through to irregular trabecular CMs and multilayering of CMs (C-E). Some *llgl1* mutants at 120 hpf exhibit apically extruding CMs (arrow, E). At 80 hpf in *llgl1* mutants, CMs extrude apically from multiple locations in the ventricular wall (arrows G-J). (K) Quantification of extruding cell number in wild-type siblings and *llgl1* mutant embryos at 55 hpf (wt, *n*=11; *llgl1^−/−^*, *n*=11), 80 hpf (wt, *n*=15; *llgl1^−/−^*, *n*=27) and 120 hpf (wt, *n*=6; *llgl1^−/−^*, *n*=19). (L) Distribution of extruding cells in wild-type and *llgl1* mutant embryos at 80 hpf. Schematic depicts the location of the outer curvature (OC), ventricular apex (VA), outflow tract (OFT) and inner curvature (IC) in the ventricle. Comparative analysis performed using one-way ANOVA (****P<0.0001). ns, non significant. Box plots show median values (middle bars) and first to third interquartile ranges (boxes); whiskers indicate 1.5× the interquartile ranges; dots indicate data points.

We next investigated ventricular wall development in *llgl1* mutants. After initiation of trabeculation at 120 hpf, *llgl1* mutants exhibit phenotypes ranging from similar trabecular organisation as wild-type (Fig. 1A,B) through to complete disorganisation of the trabeculae, including ventricular CM multilayering (Fig. 1C-E; Fig. S2U-W). We occasionally observed embryos in which CMs were extruding apically into the pericardial cavity (Fig. 1E), suggesting defects in polarity or integrity of the ventricular wall. Apically-extruding cells have been previously described at earlier stages in zebrafish embryos harbouring mutations in the epithelial-mesenchymal transition (EMT)-related gene *snai1b* (Gentile et al., 2021) or in mutants with defects in junctional protein trafficking (Grassini et al., 2019). We hypothesised that, as Llgl1 is involved in apicobasal polarity, it may play a similar role in maintaining ventricular wall integrity at earlier time points, and analysed the ventricular wall before trabeculation at 55 hpf and during early trabeculation at 80 hpf. At all stages we observed CMs that extrude from the apical surface of the ventricle into the pericardial cavity in *llgl1* mutants (Fig. 1F-K). At 55 hpf, CM extrusion in *llgl1* mutants was not significant; however, at 80 hpf we observed a significant increase in the number of extruding CMs in *llgl1* mutants compared with wild-type siblings. By 120 hpf, when trabeculation was driven by elaboration of trabecular seeds rather than CM delamination (Samsa et al, 2013), extruding cell number in *llgl1* mutants was comparable with wild type (Fig. 1K). Quantification of CM number revealed no overall changes in cell number in *llgl1* mutants compared with wild-type siblings at 55 hpf and 80 hpf (Fig. S2T), suggesting the proportion of extruding cells is not large enough to significantly alter total CM number. The small number of extruding CMs previously observed in wild-type embryos are primarily located around the ventricular apex proximal to the atrioventricular canal (Gentile et al., 2021), consistent with our analysis of extruding cell position in wild-type siblings at 80 hpf (Fig. 1L). However, extruding CMs in *llgl1* mutants are distributed throughout the ventricle, including elevated numbers in the outer curvature and ventricular apex, as well as CMs in the inner curvature and outflow tract. Together this suggests that *llgl1* is required for ventricular wall maturation.

### Loss of *llgl1* results in temporal defects in Crumbs redistribution and cardiomyocyte apicobasal polarity

**Fig. 2.**
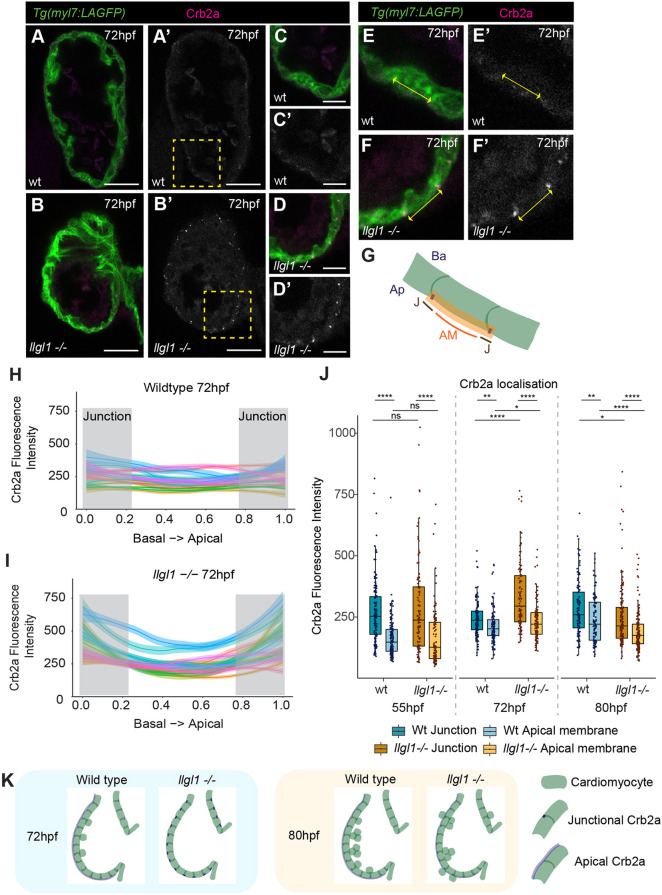
**Llgl1 is required for timely apical redistribution of Crb2a.** (A-D) Confocal *z*-slices of the ventricle of *Tg(myl7:LifeAct-GFP)* transgenic embryos visualising the myocardium (green) and anti-Crb2a antibody (magenta). (A′-D′) Crb2a staining. Scale bars: 25 µm. C and D show higher magnification of yellow boxes in A and B, respectively. Scale bars: 10 µm. In wild-type embryos at 72 hpf low levels of Crb2a are distributed across the apical myocardial membrane (A,C), whereas in *llgl1* mutants bright Crb2a puncta are observed at apical CM junctions (B,D). (E-F’) Example measurements of Crb2a in wild-type and *llgl1* mutant embryos. (G) Schematic showing quantification of Crb2a across the apical CM surface. AM, apical membrane; Ap, apical; Ba, basal; J, junction. (H,I) Example quantifications of Crb2a intensity across the standardised apical membrane of individual cardiomyocytes in wild type (H) and *llgl1* mutants (I) at 72 hpf. Crb2 is elevated at the cell boundaries in *llgl1* mutants (I) compared with wild type (H). Individual traces represent individual embryos. Grey boxes indicate apicobasal positions used to bin data into junctional domains. (J) Quantification of junctional/apical Crb2a intensity in wild-type siblings and *llgl1* mutants at 55 hpf (wt, *n*=139; *llgl1^−/−^*, *n*=113), 72 hpf (wt, *n*=117; *llgl1*^−/−^, *n*=106) and 80 hpf (wt, *n*=112; *llgl1*^−/−^, *n*=159). At 72 hpf wild-type embryos have slightly elevated Crb2a at CM junctions compared with across the apical membrane, but *llgl1* mutants have significantly more junctional Crb2a. By 80 hpf, overall levels of Crb2a in *llgl1* mutants is reduced compared with wild-types. One-way ANOVA with multiple comparisons (****P<0.0001, **P<0.01, *P<0.05). ns, non significant. Box plots show median values (middle bars) and first to third interquartile ranges (boxes); whiskers indicate 1.5× the interquartile ranges; dots indicate data points. (K) Schematic depicting dynamics of Crb2a distribution in wild type and *llgl1* mutants at 72 hpf and 80 hpf.

The ventricular wall exhibits apicobasal polarity before and during trabeculation (Jiménez-Amilburu et al., 2016; Jiménez-Amilburu and Stainier, 2019). Loss of the apicobasal polarity regulator Crb2a results in multi layering of polarised CMs, but not apical CM extrusion (Jiménez-Amilburu and Stainier, 2019). Conversely, however, improper CM trafficking of N-cadherin is associated with apical CM extrusion (Grassini et al., 2019). As the *llgl1* mutant displays both ventricular CM multilayering and extrusion, our data suggested a complex relationship between ventricular wall apicobasal polarity and organised CM delamination. We investigated whether zebrafish Llgl1 plays a role in Crb2a redistribution from apical CM junctions to the apical membrane during trabeculation (Jiménez-Amilburu and Stainier, 2019), examining Crb2a distribution across the apical CM surface in wild-type and *llgl1* mutant embryos (Fig. 2A-G). Both wild-type and *llgl1* mutant embryos exhibit clear upregulation of Crb2a at apical cell-cell junctions at 55 hpf (Fig. 2J). By 72 hpf, wild-type embryos exhibit low levels of Crb2a along the apical CM membrane at 72 hpf, with slightly higher junctional than apical Crb2a (Fig. 2H,J). Conversely, *llgl1* mutants have a strong retention of Crb2a at CM junctions compared with wild-type junctions, or compared with the apical membrane of mutant CMs (Fig. 2I,J). However by 80 hpf the junctional:apical membrane distribution of Crb2a in *llgl1* mutants has become more comparable with that observed in wild-type CMs and, in general, *llgl1* mutants have a slight decrease in overall Crb2a levels (Fig. 2J). Together this supports a role for Llgl1 in timely Crb2a protein relocalisation or deposition in ventricular CMs (Fig. 2K). To determine whether *llgl1* mutants have general defects in epithelial polarity and integrity, we analysed aPKC, which localises to the apical domain of epithelial cells. We found apical enrichment of aPKC in wild-type cardiomyocytes at 55 hpf, in line with previous reports (Merks et al., 2018), which is unaffected in *llgl1* mutants (Fig. S4). At 72 hpf aPKC is less strongly apically-enriched in either wild-type embryos or *llgl1* mutants; however, levels of aPKC are generally lower in *llgl1* mutants compared with wild-type siblings, supporting a role for *llgl1* in maintenance of ventricular CM apicobasal polarity.

### Timely establishment of a laminin sheath around the apical ventricular surface requires Llgl1

Alongside intracellular polarity complexes, the ECM is important for epithelial polarisation, in particular laminin, a major constituent of the basement membrane (Matlin et al., 2017). Studies have associated breakdown of basement membranes with cell delamination in EMT (Zeisberg and Neilson, 2009) and developmental processes such as neural crest migration (Hutchins and Bronner, 2019). It has been suggested that trabeculation is an EMT-like process (Staudt et al., 2014; Jiménez-Amilburu et al., 2016), and mutations in the EMT regulator *snai1b* results in reduced trabeculation, along with aberrant apical ventricular CM extrusion, similar to that seen in *llgl1* mutants. Together, this raised questions around the nature of basement membrane dynamics during trabeculation, the relationship between polarity and apicobasal delamination of ventricular CMs, and the role of *llgl1* in regulating these processes.

**Fig. 3.**
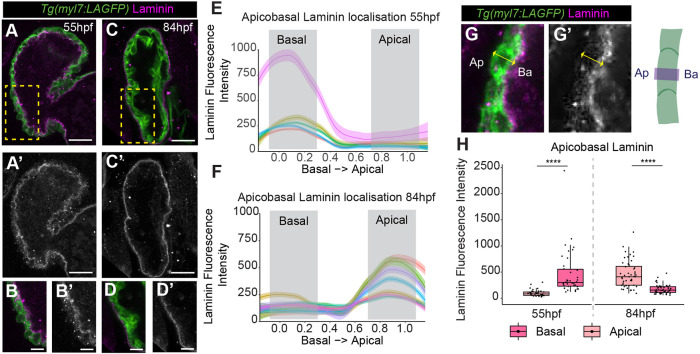
**Laminin deposition shifts from basal to apical ventricular surface.** (A-D′) Confocal *z*-slices of the ventricle of *Tg(myl7:LifeAct-GFP)* transgenic embryos visualising the myocardium (green) and anti-laminin antibody (magenta). A-D show a merge view, A′-D′ show laminin staining. Laminin is deposited on the luminal basal myocardial surface at 55 hpf (A,A′), but is enriched on the apical exterior surface of the myocardium at 84 hpf (C-D′). Scale bars: 25 µm. B and D show a magnification of the yellow boxed areas in A and C, respectively. Scale bars: 10 µm. (E,F) Example quantifications of laminin intensity across the standardised apicobasal axis of individual CMs at 55 hpf (E) and 84 hpf (F). Grey boxes indicate apicobasal positions used to bin data into apical/basal domains. (G,G′) Example images and schematic showing method for quantifying laminin across the apicobasal CM axis. Ap, apical; Ba, basal. (H) Quantification of laminin fluorescence intensity at apical and basal positions in ventricular CMs at 55 hpf and 84 hpf. Each point represents an individual cell (55 hpf, *n*=38; 84 hpf, *n*=48). One-way ANOVA with multiple comparisons (****P<0.0001). Box plots show median values (middle bars) and first to third interquartile ranges (boxes); whiskers indicate 1.5× the interquartile ranges; dots indicate data points.

To investigate basement membrane organisation, we analysed laminin deposition in zebrafish hearts before and after the onset of trabeculation. At 55 hpf, before trabecular seeding was initiated, we found laminin deposition at the luminal basal surface of ventricular CMs (Fig. 3A-B′). Once trabecular seeding was underway at 84 hpf, we observed a loss of basal laminin in ventricular CMs; however, this was unexpectedly accompanied by the deposition of laminin on the outer, apical surface of the heart (Fig. 3C-D′). We quantified the dynamics of basal laminin degradation and apical laminin establishment across the apicobasal axis of ventricular CMs (Fig. 3E-H), confirming that, at 55 hpf, laminin was only found on the basal CM surface, whereas by 84 hpf laminin had been deposited at the apical CM surface and basal laminin was absent (Fig. 3H). During trabeculation the ventricular myocardium is sandwiched between two layers of ECM, therefore we continue to refer to laminin as apical or basal in relation to the myocardium.

**Fig. 4.**
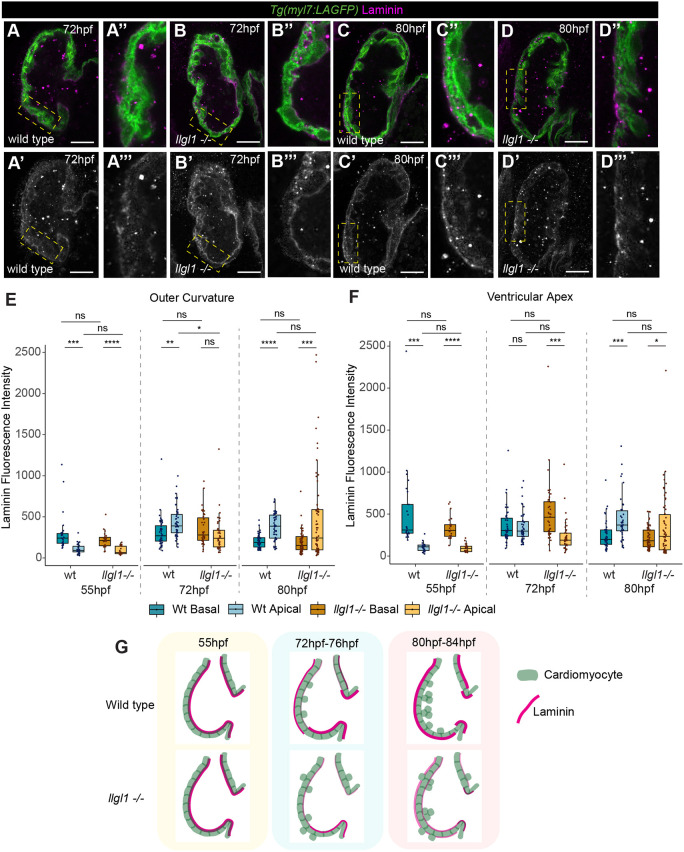
**Llgl1 promotes timely deposition of laminin to the apical ventricular surface.** (A-D‴) Confocal *z*-slices of the ventricle of *Tg(myl7:LifeAct-GFP)* transgenic embryos visualising the myocardium (green) and anti-laminin antibody (magenta) in wild-type siblings (A,C) and *llgl1* mutant embryos (B,D). Yellow boxed areas are enlarged to the right. A-D and A″-D″ show a merge view, A′-D′ and A‴-D‴ show laminin staining. *llgl1* mutants still exhibit basal laminin at 72 hpf (B), and are yet to clearly establish apical laminin at 80 hpf (D). Scale bar=25 µm. (E) Quantification of apicobasal laminin intensity in the outer curvature shows that laminin is basally enriched in both wild-type siblings and *llgl1* mutants at 55 hpf (wild-type, *n*=27; *llgl1* mutants, *n*=26). Laminin is apically enriched in wild-type siblings from 72 hpf onwards (72 hpf, *n*=48; 80 hpf, *n*=49), whereas *llgl1* mutants exhibit no apical laminin enrichment at 72 hpf (*n*=40), and only apical enrichment at 80 hpf (*n*=68). (F) Quantification of apicobasal laminin intensity in the ventricular apex reveals that both wild-type siblings and *llgl1* mutants have enrichment of basal laminin at 55 hpf (wild type, *n*=25; *llgl1* mutants, *n*=23). Wild-type siblings do not exhibit significant degradation of basal laminin and enrichment of apical laminin until 80 hpf (72 hpf, *n*=45; 80 hpf, *n*=42), whereas *llgl1* mutants still retain significant levels of basal laminin at 72 hpf (*n*=40), and do not have significant apical deposition even by 80 hpf. (E,F) Kruskal–Wallis test with multiple comparisons (*n*=58) (****P<0.0001, ***P<0.001, **P<0.01, *P<0.05). ns, non significant. Box plots show median values (middle bars) and first to third interquartile ranges (boxes); whiskers indicate 1.5× the interquartile ranges; dots indicate data points. (G) Schematic depicting dynamics of laminin distribution in wild type and *llgl1* mutants.

The timing of the basal-to-apical shift in laminin localisation in ventricular CMs coincides with the highest incidence of apical CM extrusion in *llgl1* mutants. As *llgl1* mutants had timely defects in redistribution of Crb2a, we hypothesised that *llgl1* is required for laminin deposition to the apical CM surface and that apical laminin is linked with maintaining ventricular wall integrity during trabecular seeding. We quantified apicobasal CM laminin levels in *llgl1* mutants at 55 hpf, 72 hpf and 80 hpf (Fig. 4A-D‴), separating the ventricular wall into outer curvature and ventricular apex, the regions where we observed the highest number of extruding cells (Fig. 1). At 55 hpf, *llgl1* mutants exhibited similar levels of basal laminin as wild-type embryos in both the ventricular apex and outer curvature of the ventricle (Fig. 4E,F). We found that, in wild-type embryos, laminin was already apically enriched in the outer curvature at 72 hpf (Fig. 4E), whereas at the ventricular apex, apical enrichment of laminin did not occur until ∼80 hpf (Fig. 4F), suggesting spatiotemporal regulation of laminin deposition to the apical CM surface. *llgl1* mutants had significantly less apical laminin than wild-type siblings in the outer curvature (Fig. 4E) at 72 hpf, and a significant basal retention of laminin in the ventricular apex (Fig. 4F). We started to observe apical enrichment of laminin in *llgl1* mutants only at 80 hpf (Fig. 4E,F). Levels of apical laminin in *llgl1* mutants at 80 hpf were highly variable compared with wild-type siblings, with some mutants exhibiting very high levels of apical laminin, for example in the outer curvature, whereas other mutants were yet to deposit any laminin at the apical surface. Together, this suggests that Llgl1 is required for the timely establishment of this apical laminin sheath (Fig. 4G).

### Epicardial cells deposit laminin on the apical ventricular surface

**Fig. 5.**
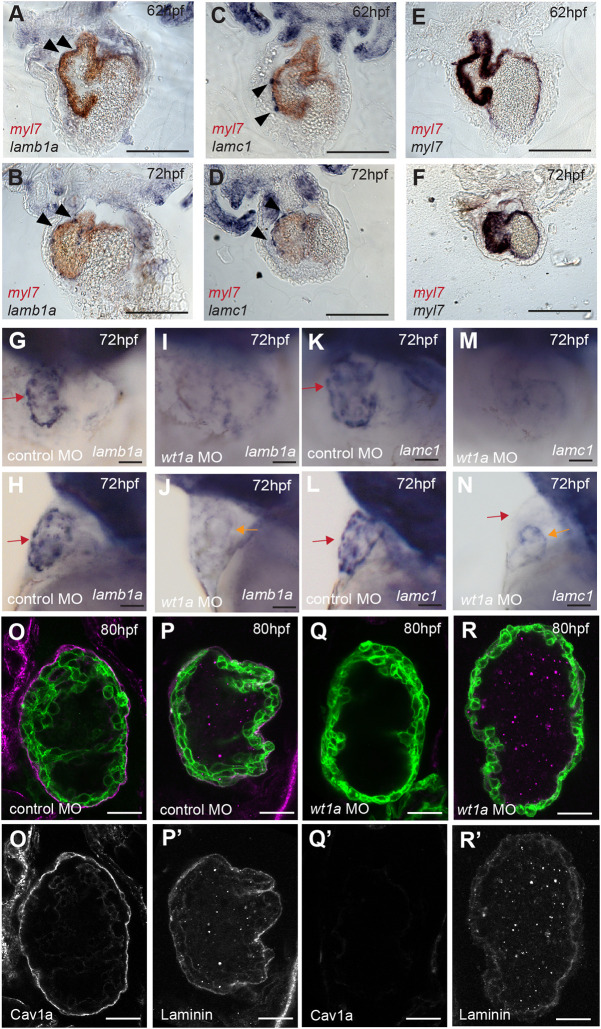
**The epicardium deposits laminin onto the apical ventricular surface.** (A-F) Sections through the hearts of two-colour mRNA *in situ* hybridisation analysis of *lamb1a* (A,B), *lamc1* (C,D) and *myl7* controls (E,F) in blue, combined with *myl7* expression (red) to highlight the myocardium, at 62 hpf and 72 hpf (*n*=3 per gene/stage). *lamb1a* and *lamc1* are expressed in epicardial cells adjacent to/on the surface of the myocardium (arrowheads), contrasting with the *myl7* blue/red control demonstrating colocalised myocardial stains. Scale bars: 200 μm. A-F show coronal sections. (G-N) mRNA *in situ* hybridisation analysis of *lamb1a* and *lamc1* expression in control MO and *wt1a* MO-injected embryos at 72 hpf. G,I,K,M show ventral views, H,J,L,N show lateral views. Scale bars: 25 µm. Control MO-injected embryos express *lamb1a* and *lamc1* around the ventricle (red arrow, *lamc1 n*=6, *lamb1a n*=5), whereas *wt1a* MO-injected embryos lose expression of both *lamb1a* and *lamc1a* around the ventricle (*lamc1 n*=9, *lamb1a n*=6) but retain low levels in the atrium (orange arrow). (O-R′) Confocal *z*-slices of the ventricle of 80 hpf *Tg(myl7:HRAS-GFP)* transgenic embryos injected with either control MO (O,P) or *wt1a* MO (Q,R), and an anti-Cav1a antibody (magenta; O,Q) or anti-laminin antibody (magenta; P,R). Scale bars: 25 µm. Control MO-injected embryos exhibit expression of Cav1a (*n*=9/10) and laminin (*n*=10/10) at the apical CM surface (O,P), whereas *wt1a* MO-injected embryos show loss of both Cav1a (Q, *n*=9/9) and laminin (R, *n*=10/10) expression. O-R show a merge view, O′-R′ show Cav1a or laminin staining.

During zebrafish heart development, epicardial coverage of the ventricle is established from ∼72 hpf (Peralta et al., 2014; Boezio et al., 2023), coinciding with laminin deposition on the ventricular surface (Fig. 3). We therefore investigated whether epicardial cells are the source of apical laminin in the ventricle, first through mRNA *in situ* hybridisation expression analysis of the major cardiac laminin subunits *lamb1a* and *lamc1* (Derrick et al., 2021a) at 62 hpf and 72 hpf (Fig. 5A-F). At 62 hpf both *lamb1a* and *lamc1* were expressed in cobblestone-like cells adjacent to the myocardium (Fig. 5A,C), with coverage increasing at 72 hpf when laminin-expressing cells had flattened out into a thin layer covering the ventricle, adjacent to *myl7-*expressing CMs (Fig. 5B,D), morphological changes reminiscent of those previously described in newly-adhering epicardial cells (Dettman et al., 1997; Pérez-Pomares and de la Pompa, 2011). This suggests that epicardial cells could be the source of laminin deposited on the apical ventricular surface. To confirm this, we injected embryos with an antisense morpholino oligonucleotide (MO) targeting *wilms tumour 1a* (*wt1a*) (Perner et al., 2007), a transcription factor expressed in the epicardium and required for epicardial development (Moore et al., 1999; Serluca, 2008; Peralta et al., 2013; Andrés-Delgado et al., 2020; Boezio et al., 2023), and assessed the impact on laminin expression and deposition. Both *lamb1a* and *lamc1* were no longer expressed around the ventricle in *wt1a* morphants (Fig. 5G-N). To confirm that the *wt1a* MO prevented epicardial development, we analysed the expression of Caveolin 1alpha (Cav1a), a structural component of caveolae expressed in epicardial cells (Grivas et al., 2020), in control MO and *wt1a* MO-injected embryos at 80 hpf. Although control MO-injected embryos had Cav1a-expressing epicardial cells (Fig. 5O), *wt1a* morphant embryos had no epicardial cells at 80 hpf (Fig. 5Q), confirming efficacy of the *wt1a* MO. Corroborating our observation that mRNA encoding laminin subunits is expressed in epicardial cells, *wt1a* morphants lost apical laminin deposition when compared with control embryos (Fig. 5P,R), confirming the epicardium as the source of laminin deposited on the ventricular surface. Conversely, *wt1a* morphants retained deposition of laminin at the basal CM surface, likely from earlier expression and deposition of laminin by either myocardial or endocardial cells (Derrick et al., 2021a; Abu Nahia et al., 2024), which was unaffected by later epicardial development.

### Llgl1 is required for timely emergence of the epicardium from the dorsal pericardium

**Fig. 6.**
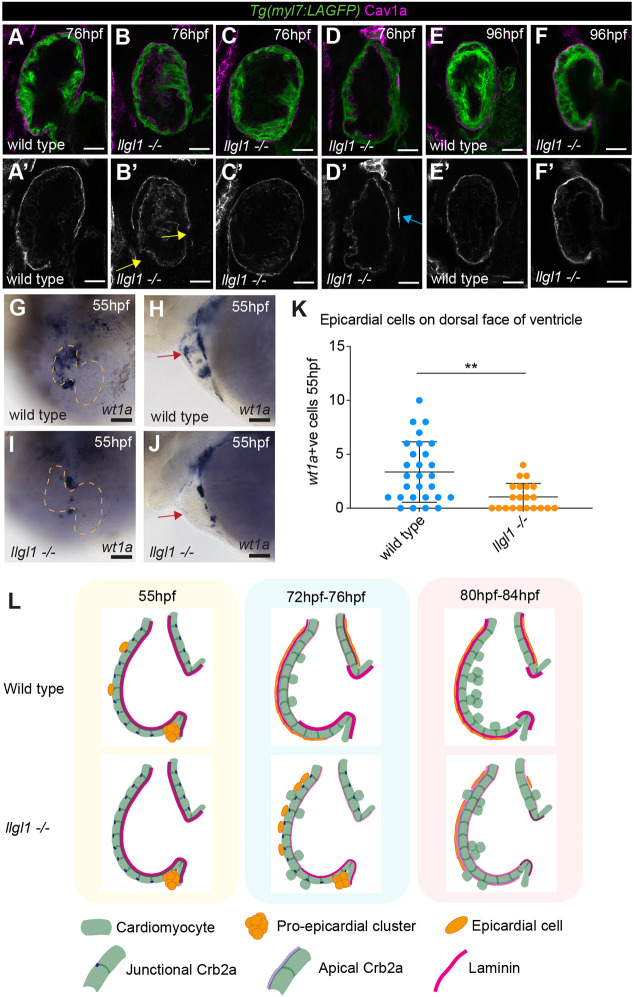
**Llgl1 is required for timely epicardial emergence and ventricular colonisation.** (A-F′) Confocal *z*-slices through the ventricle of *Tg(myl7:LifeAct-GFP)* transgenic embryos visualising the myocardium (green) and anti-Cav1a antibody (magenta), highlighting the epicardium at 76 hpf (A-D) and 96 hpf (E,F). Wild-type embryos show full epicardial coverage at 76 hpf (A, *n*=12/12), whereas only a small subset of *llgl1* mutants have full epicardial coverage (C, *n*=3/15), or only a few epicardial cells attached (D′, blue arrow, *n*=3/15) and the majority have partial epicardial coverage (B′, yellow arrows, *n*=9/15). By 96 hpf Cav1a^+^ epicardium surrounds the ventricle in both wild type (E, *n*=4/4) and the majority of *llgl1* mutant embryos (F, *n*=5/6). A′-F′ show Cav1a staining only. Scale bars: 25 µm. (G-J) mRNA *in situ* hybridisation analysis of *wt1a* expression in wild-type and *llgl1* mutant embryos at 55 hpf. G and I show the ventral view, dashed line outlines the heart; H and J show the lateral view. Scale bars: 50 µm. Both wild-type and *llgl1* mutant embryos express *wt1a* in proepicardial cells in the dorsal pericardium; however; whereas wild-type embryos have *wt1a*^+^ cells attached to the ventral ventricular wall (red arrow, H), these cells are reduced or absent in *llgl1* mutants (red arrow, J). (K) Quantification of the number of epicardial cells attached to the ventral ventricular wall in wild type (*n*=28) and *llgl1* mutants (*n*=21). Comparative analysis performed using unpaired two-tailed *t*-test (**P<0.01). Data are mean±s.d. (L) Schematic model of epicardium, laminin and Crb2a dynamics between 55 hpf and 84 hpf.

We next investigated whether *llgl1* mutants exhibit defects in epicardial attachment that could account for the delay in apical laminin establishment, analysing Cav1a in wild-type and *llgl1* mutant embryos. At 76 hpf, wild-type siblings exhibited full epicardial coverage of the ventricle (Fig. 6A). However, *llgl1* mutants displayed variability in epicardial coverage, with some mutants having either full (Fig. 6C) or almost/completely absent (Fig. 6D) epicardial coverage, whereas the majority exhibited partial epicardial coverage, often with patches of epicardium missing at the ventricular apex (Fig. 6B). However, by 96 hpf the majority of *llgl1* mutants had full epicardial coverage of the ventricle, comparable with wild-type siblings (Fig. 6E,F) suggesting that, although *llgl1* is not required for epicardial specification, it is instead required for timely dissemination of epicardial cells. To confirm this, we analysed epicardial development at 55 hpf, when proepicardial clusters are forming from the dorsal pericardium at the venous pole and atrioventricular canal and are beginning to colonise the ventricle (Andrés-Delgado et al., 2019; Boezio et al., 2023; Serluca, 2008). mRNA *in situ* hybridisation analysis of *wt1a* expression revealed that proepicardial cells are positioned in the dorsal pericardium in *llgl1* mutants, similar to wild-type siblings (Fig. 6G,I). However, although in wild-type siblings epicardial cells had begun emerging from the proepicardium and adhering to the ventral ventricular surface (Fig. 6H), in *llgl1* mutants very few epicardial cells had started colonising the ventricle (Fig. 6J,K). Previous studies have shown that cardiac contractility and hemodynamic forces are important for both ventricular wall integrity and epicardial colonisation of the ventricle (Peralta et al., 2013; Rasouli et al., 2018); however, we observed no defects in parameters associated with cardiac function in *llgl1* mutants between 55 hpf and 80 hpf (Fig. S5), suggesting that the epicardial defect in *llgl1* mutants is independent from cardiac function. Together, this supports the hypothesis that, although *llgl1* is not required for proepicardial specification, it is required for timely epicardial colonisation of the ventricle and subsequent deposition of laminin onto the apical surface of ventricular CMs (Fig. 6L).

### Laminin is required for ventricular wall integrity and epicardial development

Previous studies have identified that *wt1a* and *tcf21* mutants, which exhibit epicardial defects, display aberrant apical extrusion of CMs from the ventricular wall, supporting a hypothesis that epicardial coverage of the ventricle maintains myocardial wall integrity during trabecular seeding. However, analysis of hypomorphic alleles, in which some epicardial cells remain, suggests that the presence of epicardial cells alone is not sufficient to prevent CM extrusion (Boezio et al., 2023). Our observation that the delay in apical laminin deposition in *llgl1* mutant embryos is coincident with the stage at which aberrant CM extrusion is highest similarly suggests that establishment of an epicardially-derived apical laminin sheath around the outer surface of the ventricular myocardium may help maintain the integrity of the ventricular wall during early trabeculation. Supporting this, we analysed whether apically extruding CMs in either wild-type or *llgl1* mutant embryos were associated with less apical laminin. We quantified laminin levels across the apical membrane of both extruding cells and adjacent cells either side of the extruding cell (Fig. S6A) and, consistent with our hypothesis, we found that extruding cells in both wild-type and *llgl1* mutant embryos had significantly less laminin than their neighbours (Fig. S6B). Together, this suggests that apical laminin could maintain integrity of the ventricular wall by preventing apical cell extrusion during early trabeculation. To investigate this further, we analysed ventricular wall integrity in *lamb1a* mutants, which carry a mutation in *laminin, beta 1a*, a key subunit of cardiac laminins (Derrick et al., 2021a), which is expressed in epicardial cells (Fig. 5). Live imaging of the ventricular wall at 76 hpf revealed a significant increase in the number of apically extruding ventricular CMs in *lamb1a* mutants compared with wild-type siblings (Fig. S6C-H), albeit at lower frequency than in *llgl1* mutants. Together, this suggested that laminin is required to maintain integrity of the ventricular wall.

**Fig. 7.**
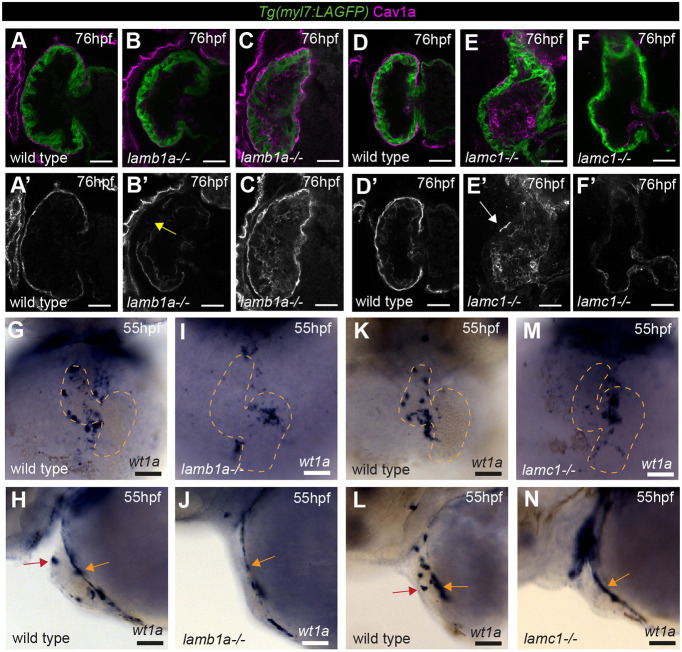
**Laminin promotes epicardial emergence and ventricular colonisation.** (A-F) Confocal *z*-slices through the ventricle of *Tg(myl7:LifeAct-GFP)* transgenic embryos visualising the myocardium (green) and anti-Cav1a antibody (magenta), highlighting the epicardium at 76 hpf in wild-type siblings (A,D), *lamb1a* mutants (B,C) and *lamc1* mutants (E,F). A′-F′ show Cav1a staining only. Scale bars: 25 µm. Wild-type embryos show full epicardial coverage at 76 hpf (A, *n*=12/12; D, *n*=11/11), whereas *lamb1a* and *lamc1* mutants show defects in epicardial coverage. The majority of *lamb1a* mutants have partial epicardial coverage (B′, yellow arrow, *n*=10/13), with a small number exhibiting full epicardial coverage (C, *n*=3/13). *lamc1* mutants have more profound epicardial defects, with the majority having no epicardial cells attached at 76 hpf (F, *n*=6/9), and a small proportion having only a few epicardial cells attached (E′, white arrow, *n*=3/9). (G-N) mRNA *in situ* hybridisation analysis of *wt1a* expression at 55 hpf in wild-type embryos (G,H,K,L), *lamb1a* mutants (I,J) and *lamc1* mutants (M,N). G,I,K,M show the ventral view, dashed line outlines the heart; H,J,L,N show the lateral view. Scale bars: 50 µm. Similar to wild-type siblings (H, *n*=10; J, *n*=12), both laminin mutants have *wt1a* expression in proepicardial cells on the dorsal pericardium (L, *n*=11; N, *n*=12; orange arrows). Wild-type embryos also have epicardial cells attached to the ventral ventricular wall (H,L; red arrows) but both *lamb1a* and *lamc1* mutants show little attachment of epicardial cells to the ventral ventricle at 55 hpf (J,N).

To confirm that we were impacting apical laminin deposition but not epicardial development we analysed Cav1a expression in *lamb1a* mutants. Surprisingly, and similar to our findings in *llgl1* mutants, the majority of *lamb1a* mutants exhibit partial epicardial coverage at 76 hpf when compared with wild-type siblings, with frequent gaps observed in the epicardium (Fig. 7A-C), suggesting that laminin is required for epicardial development. Confirming this, we also investigated Cav1a expression in *lamc1* mutants, which carry mutations in the *laminin, gamma 1* subunit (Odenthal et al., 1996; Parsons et al., 2002). *lamc1* mutants exhibit a more severe epicardial phenotype than *lamb1a* mutants, with most mutants having a complete loss of epicardial attachment to the ventricle (Fig. 7D-F). These epicardial defects in laminin mutants could result either from a failure in epicardial cells to remain adhered to the ventricular surface at 72 hpf if they are unable to produce laminin or that, similar to Llgl1, laminin is also required for epicardial emergence from the proepicardium. To investigate the latter, we analysed *wt1a* expression in *lamb1a* and *lamc1* mutants at 55 hpf and found that, similar to *llgl1* mutants, both laminin mutants exhibited defects in epicardial cell emergence (Fig. 7G-N), including retention of proepicardial cells in the dorsal pericardium and a reduction in epicardial cells positioned on the ventral ventricular surface.

Together, this suggests that, although both Llgl1 and laminin are not required for proepicardial specification, they are both required for timely epicardial colonisation, representing a broader role for apicobasal polarity proteins in epicardial emergence.

### Epicardial Llgl1 helps maintain ventricular wall integrity

We have shown that Llgl1 is required for timely deposition of laminin on the apical ventricular CM surface through regulation of epicardial emergence from the dorsal pericardium (Fig. 6L). This delayed apical laminin deposition coincides with high numbers of aberrant apically-extruding CMs in the ventricular wall (Fig. 1), suggesting that epicardial deposition of laminin helps maintain ventricle integrity during trabecular seeding. Supporting this, laminin mutants exhibit extruding ventricular CMs (Fig. S6); however, they also exhibit a similar defect in epicardial emergence as that observed in *llgl1* mutants, making it difficult to dissect whether these are independent requirements for laminin. Similarly, although zebrafish embryos lacking epicardial cells do exhibit extruding ventricular CMs, it has been suggested that this is not due to the physical presence of the epicardium itself (Boezio et al., 2023). This raises further questions about whether the CM extrusion defects in *llgl1* mutants are secondary to epicardial defects or represent a direct role for *llgl1* in CMs regulating wall integrity. *llgl1* is expressed relatively broadly throughout the embryo (Clark et al., 2012), and single cell RNA-sequencing (scRNA-seq) analysis of the developing heart at 48 hpf and 72 hpf reveal it is expressed at low levels in both myocardial and epicardial cells (Abu Nahia et al., 2024), suggesting it could play a role in both cell types.

It has been hypothesised that increased cell density in the myocardium may lead to elevated apical cell extrusion in embryos lacking epicardium (Boezio et al., 2023), and previous studies have found that increased cell density leads to delamination of trabecular CMs during trabecular seeding (Priya et al., 2020). Analysis of ventricular CM internuclear distance in *llgl1* mutants at 55 hpf and 80 hpf reveals no reduction in cell size compared with wild-type siblings (Fig. S7). To further interrogate whether myocardial *llgl1* is required to prevent aberrant CM extrusion, we generated a *Tg(myl7:llgl1-mCherry)* transgenic line, in which an Llgl1-mCherry fusion protein is expressed only in CMs. Analysis of Llgl1-mCherry localisation in CMs at 55 hpf reveals that it is enriched at the basolateral cell membrane (Fig. S8A,B), suggesting correct trafficking and localisation of the fusion protein. To determine whether myocardial Llgl1 rescues aberrant CM extrusion in *llgl1* mutant embryos, we generated wild-type, heterozygous and *llgl1^−/−^* embryos positive or negative for the *Tg(myl7:llgl1-mCherry)* transgene. However, we observed that in wild-type embryos expressing the *Tg(myl7:llgl1-mCherry)* transgene, the ventricular wall appeared to be disrupted compared with controls, with a significant increase in the number of basally-delaminating trabecular cells (Fig. S8C,D). This suggests that levels of *llgl1* in the myocardium are important in regulating trabeculation.

We then performed transplantation experiments to assess whether *llgl1* is required cell autonomously in epicardial or ventricular wall development. We injected embryos derived from an *llgl1* heterozygous incross with a fluorescent tracer, and transplanted cells from donors into non-labelled embryos derived from the same incross, resulting in dextran-labelled wild-type, heterozygous or *llgl1* mutant donor cells being transplanted into wild-type, heterozygous or *llgl1* mutant host embryos.

**Fig. 8.**
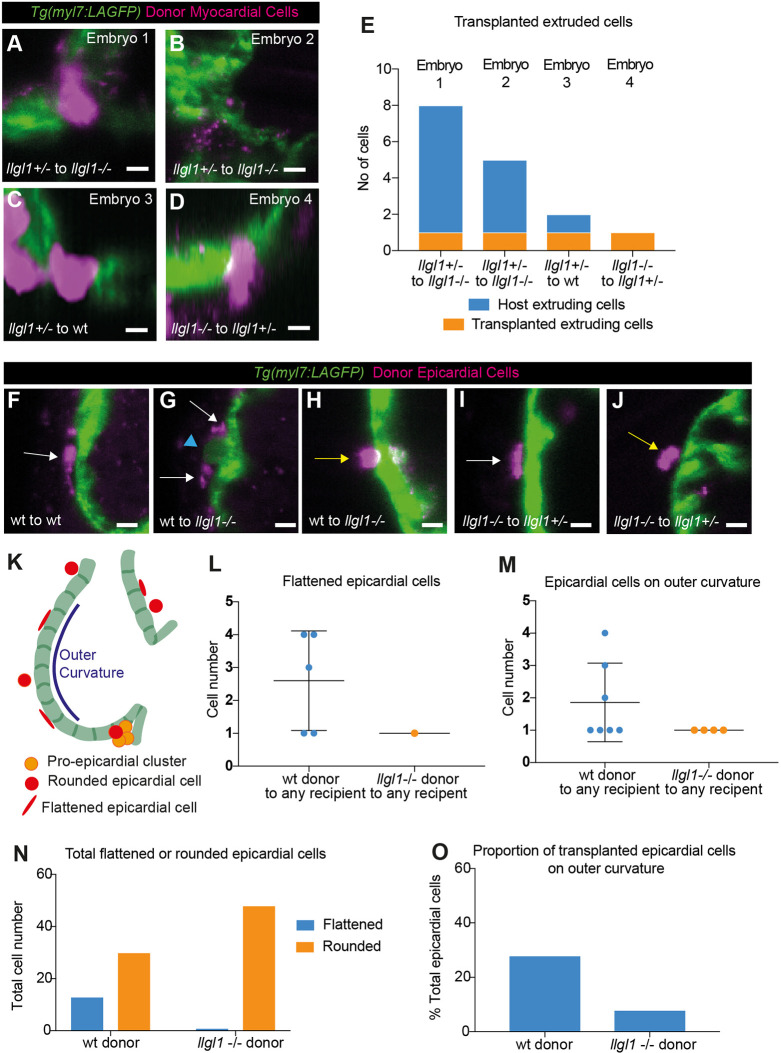
**Cell-autonomous analysis of Llgl1 requirements in epicardial colonisation and cardiomyocyte extrusion.** (A-D) Live light-sheet *z*-slices through the ventricle of *Tg(myl7:LifeAct-GFP)* transgenic embryos with rhodamine-dextran-labelled donor myocardial cells (magenta) at 76 hpf. Scale bars: 15 μm. (E) Quantification of host extruding cells and transplanted extruding cells in the four embryos with extruding transplanted CMs. (F-J) Live light-sheet *z*-slices through the ventricle of *Tg(myl7:LifeAct-GFP)* transgenic embryos with rhodamine-dextran-labelled donor epicardial cells (magenta) at 76 hpf. Scale bars: 15 μm. Transplanted epicardial cells can be flattened (white arrow, F,G,I) or rounded (yellow arrow, H,J). Epicardial cells can be seen surrounding an extruding CM (blue arrowhead, G). (K) Schematic depicting classification of epicardial cells and outer curvature. (L) Quantification of transplanted flattened mature epicardial cell number. Each dot represents a single embryo. (M) Quantification of number of epicardial cells on outer curvature. Each dot represents a single embryo. Data in L and M are mean±s.d. (N) Categorisation of total epicardial cells as flattened (mature) or rounded (immature). Flattened mature epicardial cells are significantly associated with wild-type donors (Fisher's exact test: *P*=0.0002). (O) Proportion of epicardial cells associated with the outer curvature. Wild-type transplanted epicardial cells are significantly more likely to be positioned on the outer curvature (Fisher's exact test: *P*=0.0252).

We first analysed whether transplantation of wild-type or *llgl1* mutant cells into the myocardium was associated with CM extrusion at around 76 hpf. Despite generating 51 embryos with cells transplanted into the myocardium (Table S1), including 12 embryos with *llgl1* mutant cells in wild-type/heterozygous hearts, and nine embryos with wild-type/heterozygous cells in *llgl1* mutant hearts, we only identified four embryos in which transplanted cells were extruding (Fig. 8A-E), We found that, in *llgl1* mutant hearts, heterozygous cells were able to be extruded, and that transplantation of *llgl1* mutant cells into wild-type or heterozygous hosts did not result in large numbers of extruding mutant cells, or disproportionate numbers of embryos with extruding transplanted cells – only 1/12 sibling hosts with *llgl1* mutant cells in the heart contained apically extruding mutant cells (Fig. 8E; Table S1). This supports the hypothesis that, specifically, myocardial loss of *llgl1* does not result in CM extrusion.

We next characterised and quantified transplanted cells which formed epicardium at ∼76 hpf (Fig. 8F-O), categorising donor cells as flattened and attached mature epicardial cells, or as rounded immature epicardial cells (Fig. 8K). We found that fewer *llgl1* mutant donor cells formed flattened mature epicardium compared with wild-type donor cells (Fig. 8L). Similarly, we found fewer embryos in which *llgl1* mutant donor cells had colonised the outer curvature of the ventricle (Fig. 8M), the first ventricular region colonised by epicardial cells during development (Peralta et al., 2013). Statistical analysis showed that mature epicardial cells and outer curvature epicardial cells are significantly contributed by wild-type donors (Fig. 8N,O), supporting our conclusion that Llgl1 is required in epicardial cells for timely colonisation of the ventricle. Altogether our data supports a role for Llgl1-mediated timely epicardium establishment in maintaining ventricular wall integrity.

## DISCUSSION

We describe for the first time laminin dynamics during the onset of trabeculation. Before trabeculation, laminin is deposited at the basal surface of ventricular cardiomyocytes, but at the onset of trabeculation it is degraded and an epicardially-derived sheath of laminin is deposited on the apical surface of the ventricle. Trabeculation has been described as an EMT-like process (Jiménez-Amilburu et al., 2016), and thus breakdown of laminin at the basal ventricular surface is in line with basement membrane degradation in other EMT events from embryonic development to cancer metastasis (Kalluri and Weinberg, 2009; Zeisberg and Neilson, 2009; Banerjee et al., 2022; Amack, 2021). A previous study in zebrafish has described laminin deposition at the basal surface of cardiomyocytes at 5 dpf (Marques et al., 2022), and it is possible that basal degradation of laminin is only transient to allow CM delamination associated with trabecular seeding, basal laminin being subsequently re-established.

Although not as common as basal ECM, apically-deposited ECM has been described in several biological contexts (Li Zheng et al., 2020), where it has been reported to act as external barriers on epithelia or as regulators of tissue morphogenesis. We confirmed expression of laminin subunits in the epicardium along with an epicardial requirement for apical laminin deposition. In line with this, scRNA-seq analysis at 72 hpf has identified *lamc1* as a marker of epicardial identity (Abu Nahia et al., 2024), and transcriptomic analysis of *wt1a* mutants identified a downregulation of ECM components including *lama5* (Boezio et al., 2023). We speculate that epicardially-deposited apical laminin could promote ventricular morphogenesis and help to maintain wall integrity and directionality of CM delamination during early trabeculation. It is also possible that epicardial deposition of laminin may help to anchor the epicardium to the myocardial wall after initial attachment through myocardially-derived VCAM1 (Sengbusch et al., 2002; Pae et al., 2008). We further showed that both Llgl1 and laminin are required for timely emergence of epicardial cells from the dorsal pericardium. This suggests that apicobasal polarity plays a general role in epicardial dissemination, which is in line with a previous study demonstrating a role for the PAR polarity complex member Par3 in proepicardial cyst formation (Hirose et al., 2006). It was proposed that Par3 may interpret basal cues from the underlying ECM helping to polarise proepicardial cells to form cysts, which may explain the similar epicardial defects observed in *llgl1*, *lamb1a* and *lamc1* mutants.

In addition to the roles for Llgl1 and laminin in regulating epicardial emergence, we have shown that both components help to maintain integrity of the ventricular wall, with both *llgl1* and *lamb1a* mutants exhibiting apical extrusion of ventricular CMs. Aberrant apical CM extrusion in *llgl1* mutants is unlikely to be due to disruption to initial basal laminin deposition, as this is unaffected in *llgl1* mutants. Ventricular CM extrusion has also been observed in a variety of different zebrafish mutants, including the EMT transcription factor *snai1b* (Gentile et al., 2021), the atypical myosin *myo5b*, which regulates N-cadherin trafficking in CMs (Grassini et al., 2019), the flow-dependent transcription factors *klf2a* and *klf2b* (Rasouli et al., 2018), the RA-degrading enzymes *cyp26a1* and *cyp26c1* (Rydeen and Waxman, 2016), and epicardial transcription factors *wt1a* and *tcf21* (Boezio et al., 2023)*.* The breadth of pathways implicated in CM extrusion suggests that maintenance of ventricular wall integrity is complex*.* Interestingly, these mutants also present with similar morphological defects to *llgl1* mutants, including dysmorphic ventricles and reduced looping morphogenesis. The previous analysis of hypomorphic epicardial mutants suggests that the epicardium alone does not physically restrain myocardial cells from extruding apically (Boezio et al., 2023), as there is no correlation between the number of epicardial cells on the ventricular surface and extruding CMs, and epicardial cells have been observed on extruding CMs. However, our study provides plausible evidence of an epicardial requirement for *llgl1* in preventing CM extrusion. We cannot fully account for these differences. Exploring further the likelihood of extruding CMs being directly associated with an epicardial cell, together with a granular understanding of the timing of laminin deposition post epicardial cell adhesion, may better define this relationship.

We observed a temporal delay in Crb2 relocalisation in ventricular CMs of *llgl1* mutant embryos in line with previous studies demonstrating that Lgl is required for timely apical localisation of Crumbs in *Drosophila* epithelia (Tanentzapf and Tepass, 2003). This could suggest that defects in apicobasal polarity in the ventricular wall may affect organisation of cell delamination during trabecular seeding, supporting previous studies which show that complete loss of Crb2a results in CM multilayering in the ventricular wall (Jiménez-Amilburu and Stainier, 2019). However, *crb2a* mutants do not exhibit apical CM extrusion, and unravelling the interactions between Lgl1 and Crb2a in the context of ventricular wall organisation and CM delamination may be challenging, as disrupting one of the polarity complexes can result in partial compensation from another (Tanentzapf and Tepass, 2003), which may explain the effects we observed upon expressing *llgl1* specifically in the myocardium. This impact of myocardial overexpression of *llgl1*, together with our transplantation analysis, also supports a hypothesis that the cell extrusion defects and trabeculation defects in *llgl1* mutants may arise through different mechanisms: the former due to delayed epicardial development, and the latter due to polarity defects in the ventricular wall.

Llg1 has been shown previously to be important for heart morphogenesis (Flinn et al., 2020), and although similar defects in heart morphology are apparent between the *llgl1* mutant alleles, we also observe discrepancies. Most *llgl1^sh598^* mutants are adult viable, representing a milder phenotype than the *llgl1^mw3^* allele, with which a third of mutants do not survive to adulthood; however, we found that some adult *llgl1^sh598^* mutants respond poorly to anaesthesia, suggesting cardiovascular deficiencies. Conversely, we observed severe maternal effects of the *llgl1^sh598^* mutation, with maternal-zygotic *llgl1^sh598^* mutants exhibiting variable, but often severe, defects in blastula integrity (data not shown), which are not reported in *llgl1^mw3^* mutants. These discrepancies may result from differences in genetic background that amplify specific phenotypes.

*LLGL1* lies within the Smith-Magenis microdeletion syndrome (SMS) region on human chromosome 17 (Rinaldi et al., 2022). Although the most penetrant symptoms of SMS such as sleep disorders are associated with heterozygous loss of *RAI1*, linkage studies suggest that loss of other genes within the deletion region contribute to aspects of the syndrome (Girirajan et al., 2006; Edelman et al., 2007). In particular, 20-40% of SMS patients have heart defects, and we speculate that *LLGL1* may be of importance in this syndrome as, to-date, no candidate gene has been identified that may underlie these cardiac defects (Onesimo et al., 2021). We further observed cardiac defects upon overexpression of *llgl1* in CMs. Interestingly, Potocki-Lupski syndrome, a reciprocal microduplication syndrome in which the SMS region is duplicated (including *Lgl1*) is also associated with structural heart defects (Sanchez-Valle et al., 2011; Yusupov et al., 2011; Bi et al., 2002), suggesting that levels of *Lgl1* are important in supporting heart development.

Together, our study reveals for the first time the existence of an epicardially-derived layer of laminin on the apical surface of the ventricle during heart development, providing further evidence that the epicardium helps maintain integrity of the ventricular wall during early trabeculation.

## MATERIALS AND METHODS

### Zebrafish maintenance

The following previously described lines were used: AB; *Tg(myl7:lifeActGFP)* (Reischauer et al., 2014); *Tg(fli1a:AC-TagRFP)^sh511^* (Savage et al., 2019), *Tg(-5.1myl7:DsRed2-NLS)^f2^* (Rottbauer et al., 2002); *lamb1a^sh590^* (Derrick et al., 2021a) and *lamc1^sa379^* (Kettleborough et al., 2013). Embryos were maintained in E3 medium (5 mM NaCl, 0.17 mM KCl, 0.33 mM CaCl_2_, 0.33 mM MgSO_4_) at 28.5°C and were staged as per standard protocols (Kimmel et al., 1995). Embryos older than 24 hpf were transferred into E3 medium containing 0.003% 1-phenyl 2-thiourea (PTU; Sigma-Aldrich, P7629). Animal work was approved by the local Animal Welfare and Ethical Review Body (AWERB) at the University of Sheffield, conducted in accordance with UK Home Office Regulations under PPLs 70/8588 and PA1C7120E, and in line with the guidelines from Directive 2010/63/EU of the European Parliament on the protection of animals used for scientific purposes.

### Generation of the *llgl1* mutant line

The *llgl1* mutant zebrafish line was generated using a CRISPR guide RNA designed to target Exon 2 of *llgl1* (ENSDART00000003511.11). A CRISPR target sequence (5′-GGCTATTGGAACTAAATCAGGGG-3′) in Exon 2 was identified using CHOPCHOP (Labun et al., 2016; Montague et al., 2014) and the reverse complement inserted into an ultramer scaffold as previously described (Hruscha et al., 2013) for T7 amplification: (5′-AAAGCACCGACTCGGTGCCACTTTTTCAAGTTGATAACGGACTAGCCTTATTTTAACTTGCTATTTCTAGCTCTAAAAC**CTGATTTAGTTCCAATAGCC**CTATAGTGAGTCGTATTACGC-3′; bold text indicates CRISPR site within ultramer scaffold). The ultramer was amplified by PCR (F: 5′-GCGTAATACGACTCACTATAG-3′; R: 5′-AAAGCACCGACTCGGTGCCAC-3′) and used as a template for *in vitro* transcription using MEGAshortscript T7 kit (Ambion/Thermo Fisher Scientific, AM1354). Then 2 ng gRNA was injected together with 1.9 nM Cas9 protein (New England Biolabs, M0386T) and 10% Phenol Red (Sigma-Aldrich, P0290) into the yolk at the one-cell stage, and injected F0 embryos were raised to adulthood. F0 founders were outcrossed to wild type, and resulting embryos genotyped by PCR to amplify the region of *llgl1* targeted for mutagenesis (F: 5′-GTCGGGATTGCTCTGAATAGAT-3′; R: 5′-AAGGATACATTTTGATGGCCC-3′), with mutations analysed by Sanger sequencing. A 32 bp coding sequence deletion allele was recovered, designated *llgl1^sh598^*, with the deletion region indicated by brackets: 5′-TATGATCCCA[AACTGCAGCTTATGGCTATTGGAACTAAATCA]GGGGCCATCAAAAT-3′. The deletion generates a premature stop codon in exon 2. F0 founders transmitting this mutation were outcrossed to *Tg(myl7:LifeActGFP)* and their offspring raised to adulthood. Phenotypic analyses were carried out on embryos generated from F2 or F3 adults.

### Generation of the *Tg(myl7:llgl1-mCherry)* transgenic zebrafish line

The *llgl1* full coding sequence (minus the termination codon) was amplified from wild-type cDNA using the following primers: F: 5′-ATGATGAAGTTTAGGTTCAGACGGC-3′; R: 5′-TCAGTTGATGAGGATTCCAGCAGAT-3′. This PCR product was further amplified using primers containing AttB sequences for Gateway cloning at the 5′ end of both primers, and a Kozak sequence before the initiating methionine in the forward primer (F: 5′-GGGGACAAGTTTGTACAAAAAAGCAGGCTTCGCCGCCACCATGATGAAGTTTAGGTTCAGACGGCAGGGAAATGACCCTCATCGT-3′; R: 5′-GGGGACCACTTTGTACAAGAAAGCTGGGTTTGATCCTCCTCCTCCTGATCCTCCTCCTCCGTTGATGAGGATTCCAGCAGAT-3′). The resulting PCR product was ligated into the pDONR221 middle entry Gateway vector, generating a pME-*llgl1*CDS vector. A p3E-noATGmCherry entry vector was generated by PCR-amplifying the mCherry sequence using the following primers: F: 5′-GGGGACAGCTTTCTTGTACAAAGTGGTCGTGAGCAAGGGCGAGGAGGATAACA-3′; R: 5′-GGGGACAACTTTGTATAATAAAGTTGTTTACTTGTACAGCTCGTCCATGCCG-3′. The resulting PCR product was cloned into the pDONRP2R-P3 entry vector (Kwan et al., 2007). *llgl1*CDS was subsequently recombined with the p5E:*myl7-*promoter entry vector (Veerkamp et al., 2013) and the p3E-noATGmCherry entry vector into the pDestTol2pA3 destination vector (Kwan et al., 2007) to generate the *pDestmyl7:llgl1-mCherry* construct. Gateway cloning was performed using the Tol2kit via standard protocols (Kwan et al., 2007). Then 10 pg of *pDestmyl7:llgl1-mCherry* was co-injected with 20 pg of *tol2* mRNA into the cell of one-cell-stage wild-type embryos. At 3 dpf, healthy embryos displaying myocardial mCherry expression were selected and grown to adulthood. Founder F0 fish were identified by outcrossing and the progeny (F1) displaying the brightest expression were grown to adulthood. The transgenic line was established from F1 adults displaying a Mendelian ratio of transgene transmission. The transgenic line is designated *Tg(myl7:llgl1-mCherry)^sh679^*.

### Immunohistochemistry

Embryos were fixed in 4% paraformaldehyde (PFA) with 4% sucrose either overnight at 4°C or for 3 h at room temperature, and subsequently washed into methanol. After rehydration, embryos were blocked in 0.1% PBS-Triton X-100 with 10% goat serum. Embryos were incubated overnight at 4°C with primary or secondary antibody in blocking solution. The following antibodies were used: anti-aPKC (Santa Cruz Biotechnology, sc-216, 1:100), anti-Caveolin1a (Cell Signaling Technology, D46G3, 1:100); anti-Crb2a (ZIRC, zs-4, 1:50); anti-GFP (Aves labs, GFP-1020, 1:500); anti-DM-Grasp (Developmental Studies Hybridoma Bank, zn-8-s, 1:100); anti-laminin (Sigma-Aldrich, L9393, 1:100); donkey anti-chicken-Cy2 (Jackson ImmunoResearch, 703-225-155, 1:200); goat anti-rabbit-Cy3 (Jackson ImmunoResearch, 111-165-003, 1:200); goat anti-mouse Cy5 (Jackson ImmunoResearch, 115-175-166, 1:200). Following removal of the secondary antibody, embryos were dissected, mounted in Vectashield, and imaged on a Nikon A1 confocal microscope at 40× magnification with a 1 µm *z*-slice interval.

### Morpholino oligonucleotide-mediated gene knockdown

Wt1a was depleted using a previously published splice-blocking *wt1a* MO targeting the first splice site of the *wt1a* gene (sequence: AAAGTAGTTCCTCACCTTGATTCCT) (Perner et al., 2007). The standard control MO targeting human beta-globin intron mutation was used as a negative control (GeneTools, sequence: CCTCTTACCTCAGTTACAATTTATA). All MOs were supplied by GeneTools and diluted to a 1 mM stock in MilliQ water (Millipore). Working concentrations were as follows: *wt1a*, 200 nM; control, 200 nM. Embryos were injected with 1 nl of MO solution.

### *In situ* hybridisation

Embryos were fixed overnight in 4% PFA, washed in PBST (phosphate buffered saline with 0.1% Tween) and transferred stepwise into 100% methanol for storage at −20°C. mRNA *in situ* hybridization was carried out as previously described (Noël et al., 2013). Previously published mRNA *in situ* hybridisation probes are as follows: *lamb1a* and *lamc1* (Derrick et al., 2021a), *wt1a* (Bollig et al., 2006), *myl7* (Yelon et al., 1999). Riboprobes were transcribed from a linearized template in the presence of DIG-11-UTP or Fluorescein-11-UTP (Roche). To analyse myocardial versus epicardial gene expression, stained embryos were transferred from methanol to a 30% sucrose solution and agitated overnight. The samples were then frozen in a 1 cm mould using O.C.T. Embedding Matrix for Frozen Sections (Pyramid Innovation, R40020-E) on a copper plate cooled with liquid nitrogen.

### Quantification of heart function

Embryos were transferred in batches of four (two siblings, two mutants) into E3 and 4.2% tricaine from a 28.5°C incubator to a dissection microscope attached to a high-speed camera (Chameleon3 USB3, FLIR Integrated Imaging Solutions). The heart of each embryo was located and image sequences (.tif) were captured for up to 20 s at 150 frames per second using SpinView Software (Spinnaker v. 2.0.0.147). Image sequences were imported into Fiji and a line drawn through the heart, from which a kymograph was generated to visualise periodicity of cardiac contraction. Heart rate was quantified from kymographs. Individual values represent an average heart rate over a 60 s period. Ventricular shortening was calculated from the ratio of systolic/diastolic ventricular width. Systolic fraction was calculated from the ratio of systolic/diastolic area. Blood flow was measured as distance of blood cell movement in the tail vein of the zebrafish/20 s.

### qPCR

RNA was Trizol extracted from 50×80 hpf embryos for each group, and cDNA generated using SuperScript IV Reverse Transcriptase (Invitrogen). Primers used for the qPCR were: *llgl1* primer set 1 (F: 5′-CGCTGTGTGGAGTGGATATAG-3′; R: 5′-CTTGTGACTTGTGTGTTCCATTAG-3′); *llgl1* primer set 2 (F: 5′-CCCAGACTTGGAATCCAGAAA-3′; R: 5′-CTCATCACCAAGAACCATGACTA-3′); *ef1a* (F: 5′-TCATCAAGAGCGTTGAGAAGAA-3′; R: 5′-AACGGTGTGATTGAGGGAAA-3′); *scl25a5* (F: 5′-CTGGGTAACTGCTTGGTAAAGA-3′; R: 5′-CGAAGTAGGCAGCTCTGTAAAT-3′). qPCR was performed using HOT FIREPol EvaGreen qPCR Mix Plus (no ROX) (Thistle Scientific) on a QuantStudio 12K Flex Real-Time PCR System (Applied Biosystems). All reactions were performed in triplicate with five different wild-type samples and five paired *llgl1^−/−^* samples. Relative fold change in *llgl1* mutant samples was calculated against the paired wild-type group. Statistical significance was analysed using the Wilcoxon signed rank test.

### Transplantation

Fertilised eggs were collected from an *llgl1^+/−^ Tg(myl7:LifeActGFP)* incross and donor cells were injected at the one-cell stage with 5 ng rhodamine b isothiocyanate-dextran (Sigma-Aldrich, R8881, 100 mg). Age-matched siblings were set aside as recipient embryos. At 3.5 hpf, embryos were manually dechorionated in Chorion media (pH 7.2) supplemented with penicillin/streptomycin. Using a microforge-polished transplantation needle, rhodamine dextran^+^ cells aspirated from a donor blastula were transplanted to the blastula margin of a recipient between 4 and 5 hpf. The remaining donor embryo was transferred into DNA extraction buffer immediately for genotyping. Transplanted embryos were raised individually in a 24-well plate to ∼80 hpf. The hearts of recipients that had developed normally were imaged using the Zeiss Z1 light-sheet microscope, and subsequently genotyped.

### Live light-sheet imaging

Embryonic zebrafish hearts were imaged live using a Zeiss Z1 light-sheet microscope. The embryos were anaesthetised, and heart contractility arrested by incubating the embryos in 8.4% tricaine in E3 at 10°C. *Z*-stacks encompassing the entire heart were acquired as previously described (Derrick et al., 2021b).

### Adult zebrafish swimming endurance analysis

Adult zebrafish exercise tolerance was determined by testing swimming endurance of 6-month-old zebrafish in a custom-built swim tunnel (Chapman et al., 2013) and calculating the UCRIT (critical swimming speed). UCRIT is defined as the maximum velocity the fish can maintain for a set period following the methodology described in Ramesh et al. (2010). The experiment was repeated on the same fish over three separate weeks and the average UCRIT per fish calculated. At the end of the experiment the experimental fish were culled, photographed and weighed, to calculate the UCRIT normalised to weight.

### Image quantification

Before quantification, image files were anonymised using an ImageJ Blind_Analysis plugin (modified from the Shuffler macro, v1.0 26/06/08, Christophe Leterrier, Aix-Marseille University, France). Fluorescent signal intensities were measured using ImageJ. Looping ratio was quantified as previously described (Derrick et al., 2021a) by dividing the looped distance between the two cardiac poles by the linear distance. Extruding cell number was quantified by manually inspecting each *z*-stack and defined as a cell projecting from the apical surface sufficient to perturb the contour of the external ventricular wall.

Apical laminin deposition was quantified by measuring laminin fluorescence intensity across the apicobasal axis of myocardial cells, based on a previously described method (Gentile et al., 2021). *myl7:LifeActGFP* signal was also quantified to define the apicobasal geometry of each cell, a threshold was set for GFP intensity that defined the apicobasal limits of the cell. This signal was used to coerce all cells to a uniform width with a range of 0 (basal) to 1 (apical), standardising cell size and facilitating relative analysis of laminin distribution at basal or apical sites irrespective of cell size. Laminin signal intensity was binned to basal (−0.05 to 0.3) and apical (0.7 to 1.05) regions of each cell, allowing comparison across regions and genotypes. Each cell represents an individual experimental unit.

Junction or apical membrane Crb2a and aPKC localisation was quantified by drawing a 5 px thick line across the apical surface of individual CMs, from one cell-cell junction to another, using the *myl7:LifeActGFP* signal as a landmark for cell-cell junctions. Similar to the process described for laminin, cells were coerced to the same geometry of width (0 to 1), and the Crb2 signal was binned to junctional (0 to 0.2 and 0.8 to 1) and apical (0.2 to 0.8) membrane regions of each cell. Each cell represents an individual experimental unit.

Heart size, myocardial tissue volume and lumen volume were computed by first performing live light-sheet imaging of hearts carrying the *Tg(myl7:lifeActGFP)* and *Tg(fli1a:AC-TagRFP)* transgenes, to generate 3D *z*-stacks in which the myocardium is labelled green and the endocardium red. These 3D stacks were then pre-processed and analysed using the morphoHeart software as previously described (Sánchez-Posada and Noël, 2024 preprint).

Internuclear distance was computed by first performing live light-sheet imaging of hearts carrying the *Tg(myl7:lifeActGFP)* and *Tg(-5.1myl7:DsRed2-NLS)^f2^* transgenes to generate 3D *z*-stacks in which the myocardium is labelled green and myocardial nuclei red. Nuclei coordinates were generated using the Imaris spot detection tool. Nuclei coordinates were imported into morphoCell (Sánchez-Posada and Noël, 2024 preprint), in which ventricular nuclei were annotated, non-overlapping nuclear clusters comprised a central seed cell and four nearest neighbouring cells, and the Euclidean distance between the central seed cell and its neighbouring cells in each cluster automatically measured and averaged, then averaged for all ventricular clusters per embryo.

Statistical analysis of quantitative data was performed in GraphPad Prism 9 or using the Statix package in R. Data were tested for normality to determine the most appropriate statistical test. Data were considered significant when *P*<0.05.

## Supplementary Material



10.1242/develop.202482_sup1 Supplementary information
