# Supplementary material for: Llgl1 mediates timely epicardial emergence and establishment of an apical laminin sheath around the trabeculating cardiac ventricle
Source: Development. 2024 Jun 28;151(13):dev202482. doi: 10.1242/dev.202482 (PMC11234374; doi:10.1242/dev.202482)
Supplement: Supplementary information [file develop-151-202482-s1.pdf]

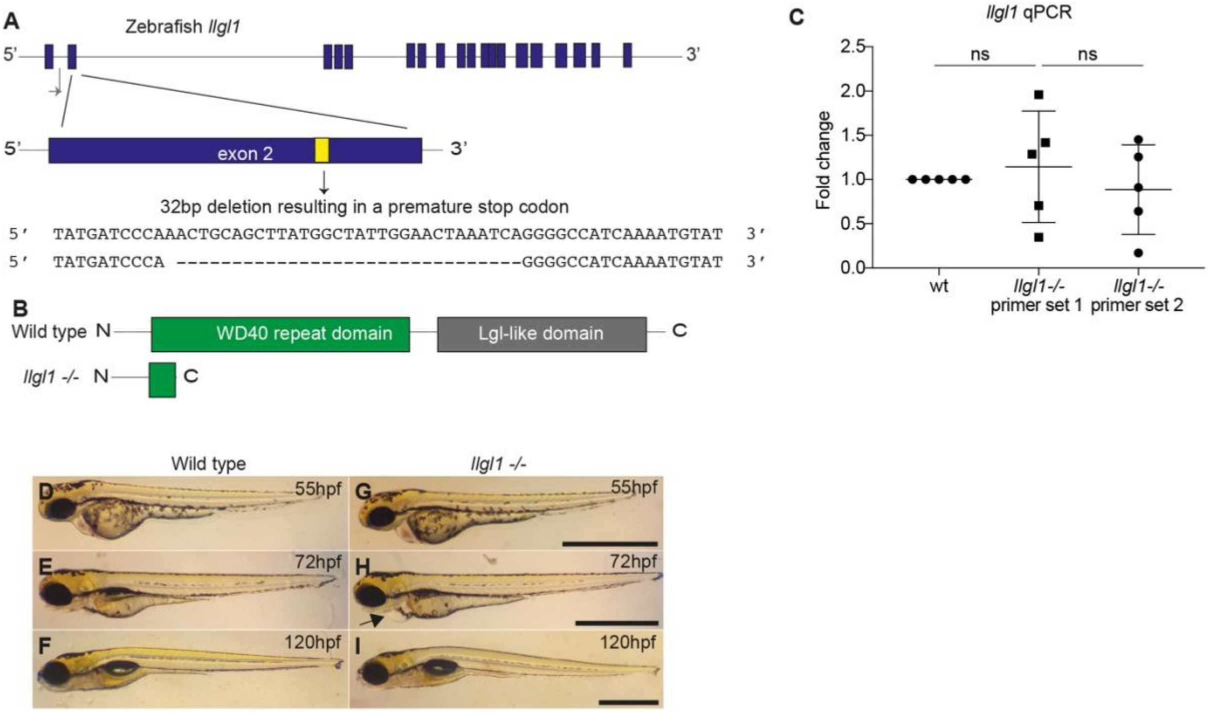

Fig. S1. Generation of an *llgl1* zebrafish mutant

A: CRISPR-Cas9-mediated mutagenesis targeting exon2 of zebrafish *llgl1* resulted in a 32bp deletion in exon 2. B: The 32bp *llgl1* deletion results in a premature stop codon generating a truncated protein lacking all functional domains of Llgl1. C: qPCR analysis of *llgl1* transcipt levels in *llgl1* mutants, using primer sets targeting two regions of the *llgl1* gene. No significant difference in *llgl1* expression was observed in *llgl1* mutants compared to wild type (T-test, ns = non significant). D-I: Brightfield images of *llgl1* mutants, lateral view, anterior to left. Scale bar = 1mm. *llgl1* mutants exhibit mild cardiac oedema at 72hpf (arrowhead, G) which resolves in most embryos by 5dpf (H).

Development • Supplementary information

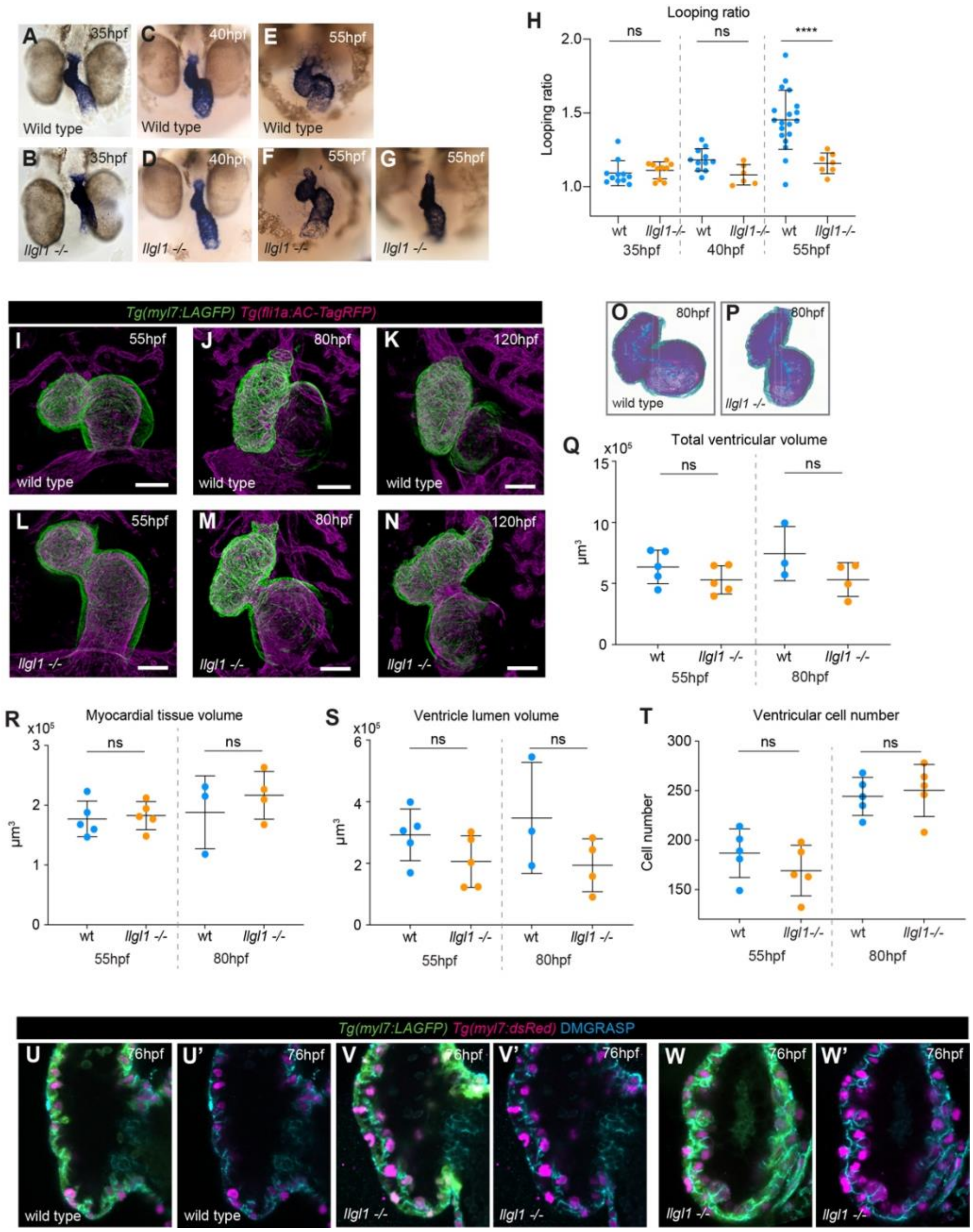

Fig. S2. ***llgl1*** **mutants have defects in heart morphology and ventricular wall organisation.**

A-H: Analysis of heart looping morphogenesis in wild type, heterozygotes, and *llgl1* mutant embryos between 35hpf and 55hpf using mRNA *in situ* hybridisation analysis of *myl7* expression and quantification of looping ratio (H, see Materials and Methods for details). At 35hpf and 40hpf, there is no difference in looping ratio between genotypes (35hpf: wt n=16; *llgl1* -/- n=14; 40hpf: wt n=13; *llgl1* -/- n=10). At 55hpf *llgl1* mutants have a significant looping defect (wt n=31; *llgl1* -/- n=22). Heart morphology in *llgl1* mutants at 55hpf is variable, with some mutants exhibiting milder looping disruption (F), while others exhibit severe

Development • Supplementary information

morphological defects (G). I-N: live lightsheet microscopy of *Tg(myl7:LifeAct-GFP);Tg(fli1a:AC-TagRFP)* double transgenic wild-type and *llgl1* mutant embryos, highlighting the myocardium (green) and endocardium (magenta). *llgl1* mutants exhibit ongoing defects in heart morphogenesis from 55hpf when compared to wild-type siblings. Scale bar = 50µm. O-S: Morphological analysis of wild type and *llgl1* mutant embryos using morphoHeart software (O, P) at 55hpf and 80hpf. *llgl1* mutants do not exhibit defects in total ventricle volume, ventricular myocardial volume, or lumen volume. T: Quantification of cardiomyocyte number in *llgl1* mutants and wild type embryos at 55hpf and 80hpf. No significant differences in cell number are observed. U-W: Confocal slices through the heart of wild type (U) and *llgl1* mutant embryos (V,W) at 76hpf, showing the myocardium (green), myocardial nuclei (magenta), and myocardial membranes (cyan). *llgl1* mutant embryos exhibit multicellular layering in the ventricular wall. H: Kruskal-Wallis test, Q-T One-way ANOVA. **** $p<0.0001$, ** $p<0.01$.

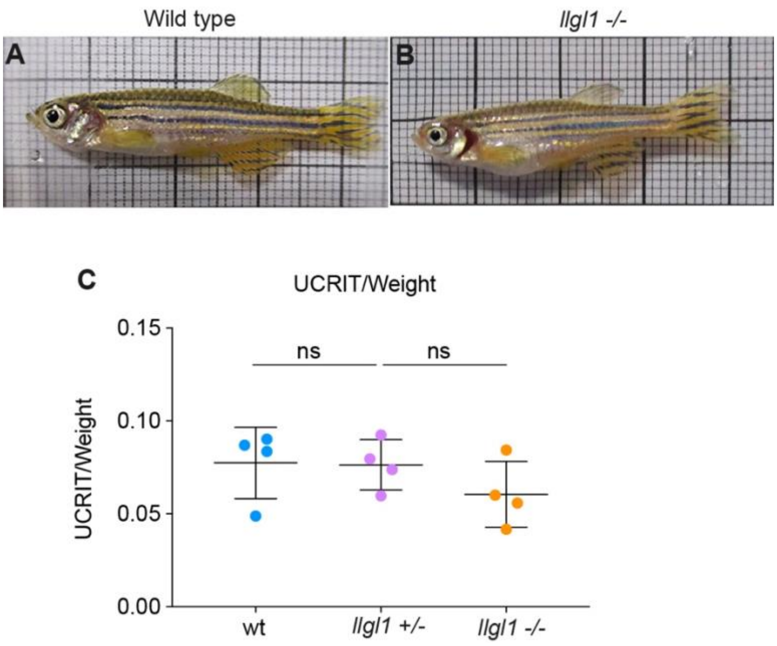

Fig. S3. *llgl1* mutants are adult viable

A-B: Brightfield images of adult wild type and *llgl1* mutant zebrafish. C: Swim tunnel performance of wild type, *llgl1 +/-* and *llgl1* mutant adult zebrafish. No difference in UCRIT was observed between groups. One-way ANOVA, ns = non significant.

Development • Supplementary information

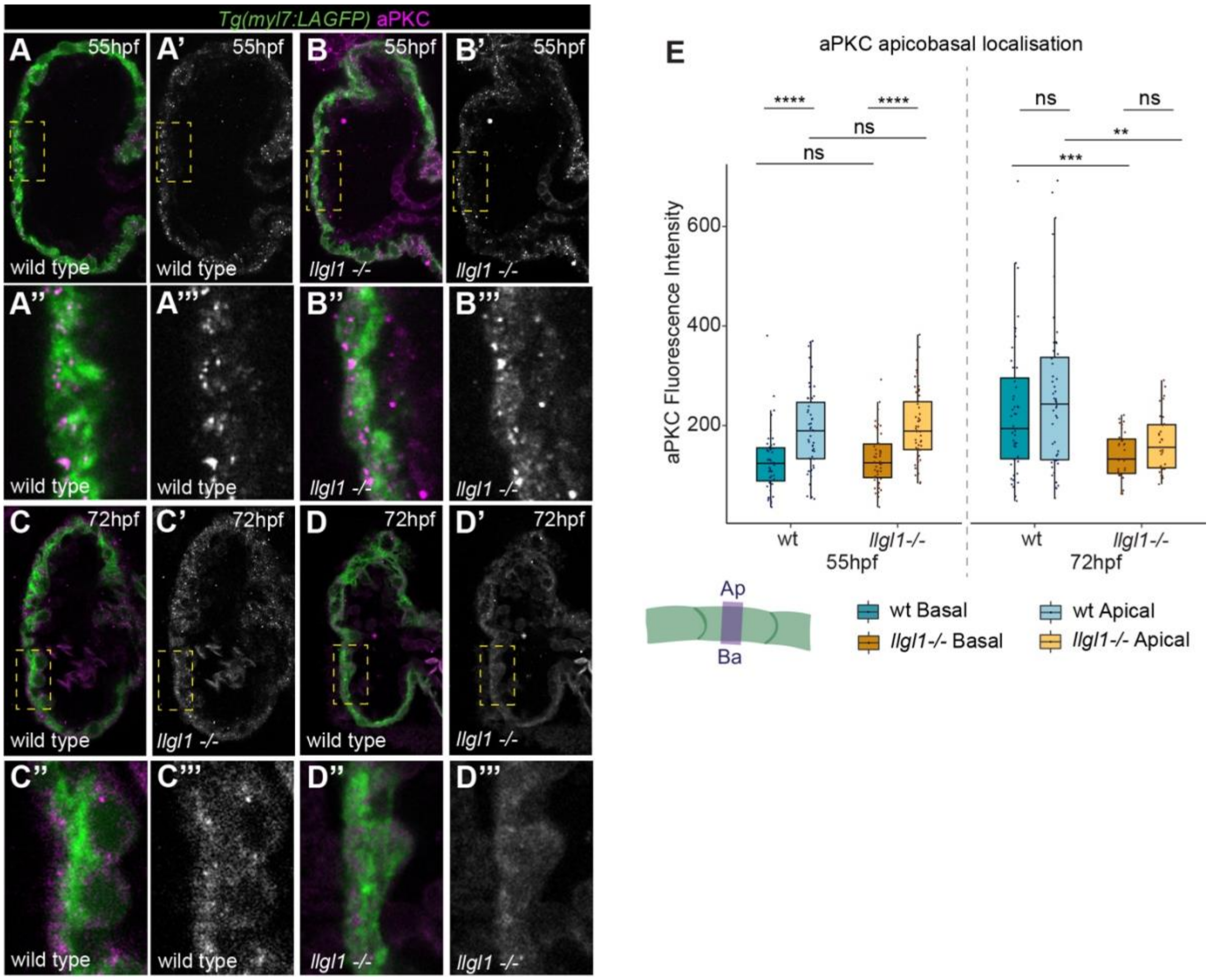

Fig. S4. Llgl1 is required for aPKC levels in cardiomyocytes at 72hpf

A-D: Confocal z-slices of the ventricle of *Tg(myl7:LifeAct-GFP)* transgenic embryos visualising the myocardium (green) and anti-aPKC antibody (magenta) in wild-type siblings (A,C) and *llgl1* mutant embryos (B,D) at 55hpf (A,B) and 72hpf (C,D). Yellow boxed areas are enlarged.. A-D, A’’-D’’: merge, A’-D’, A’’’-D’’’: aPKC. E: Quantification of basal:apical aPKC intensity in wild-type siblings and *llgl1* mutants at 55hpf, and 72hpf. At 55hpf both wild type and *llgl1* mutants have elevated aPKC at the apical surface of CMs, but by 72hpf this elevation is no longer significant. Levels of aPKC are comparable between genotypes at 5hpf, but slightly reduced in *llgl1* mutants compared to wild type siblings at 72hpf.

One-way ANOVA with multiple comparisons, **** p<0.0001, *** p<0.001, ** p<0.01, ns = non significant.

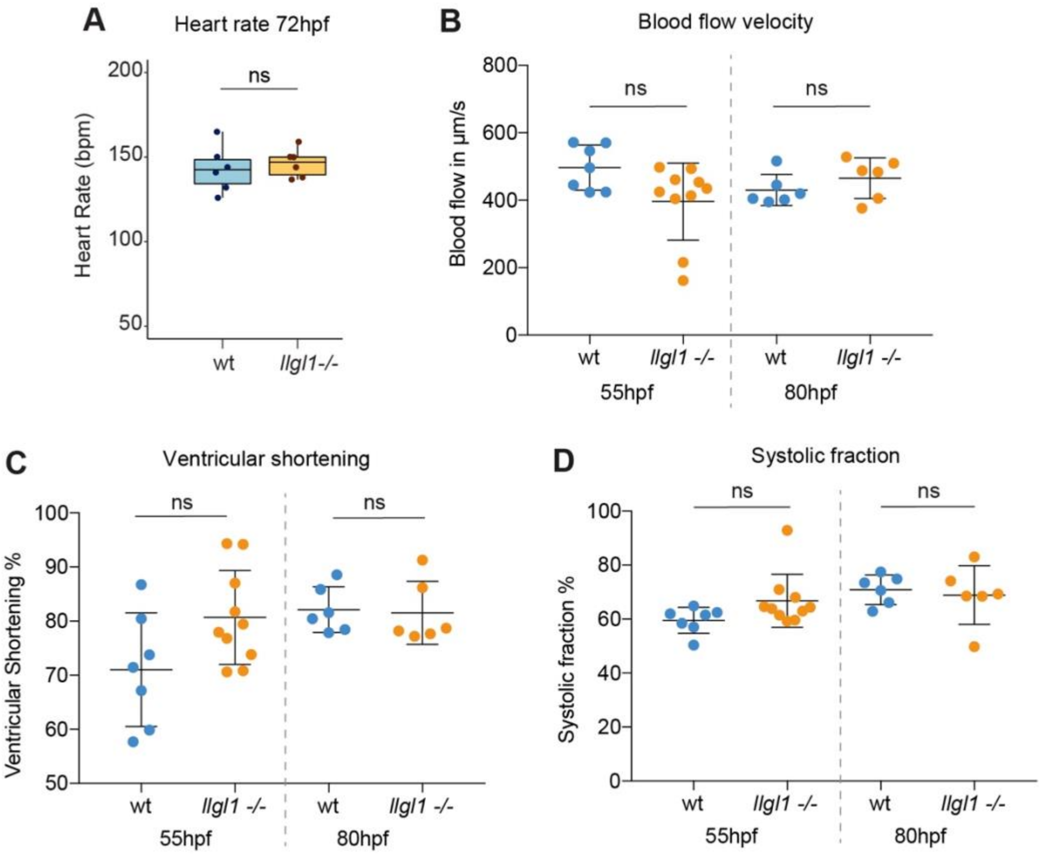

Fig. S5. Cardiac function is unaffected in *llgl1* mutants.

A: Quantification of heart rate in wild-type siblings (n=6) and *llgl1* mutants (n=6) at 72hpf. No significant difference in heart rate is observed in *llgl1* mutants. B: Quantification of blood flow velocity in the dorsal aorta of wild-type siblings (55hpf: n=7; 80hpf: n=6) and *llgl1* mutants (55hpf: n=10; 72hpf: n=6). No significant difference in blood flow velocity is observed in *llgl1* mutants at either 55hpf or 80hpf. C: Quantification of ventricular shortening in wild-type siblings (55hpf: n=7; 80hpf: n=6) and *llgl1* mutants (55hpf: n=10; 72hpf: n=6). No significant difference in ventricular shortening is observed in *llgl1* mutants at either 55hpf or 80hpf. D: Quantification of ventricular systolic fraction in wild-type siblings (55hpf: n=7; 80hpf: n=6) and *llgl1* mutants (55hpf: n=10; 72hpf: n=6). No significant difference in systolic fraction is observed in *llgl1* mutants at either 55hpf or 80hpf. T-test, ns = not significant.

Development • Supplementary information

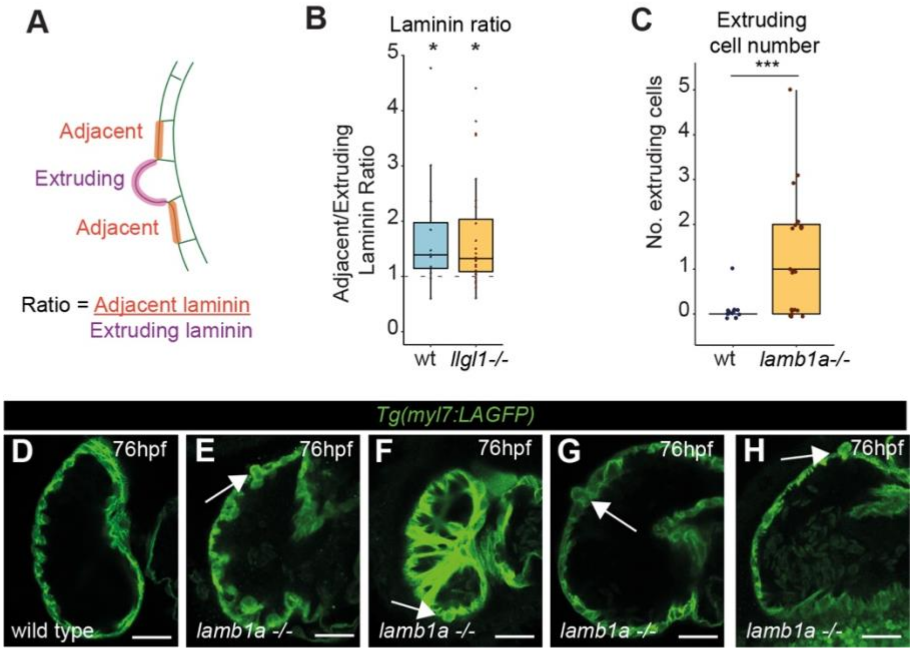

Fig. S6. *lamb1a* mutants exhibit apical ventricular CM extrusion.

A: Schematic depicting quantification of adjacent/extruding Laminin ratio. B: Quantification of adjacent cell/extruding cell Laminin ratio in both wild-type sibling and *llgl1* mutant embryos. A value of greater than 1 indicates lower apical Laminin in extruding cells than adjacent cells. 1-sided T-test, * p<0.05. C: Quantification of extruding cell number at 80hpf in wild-type siblings (n-13) and *llgl1* mutant embryos (n=22). T-test, *** p<0.001. D-H: Confocal z-slices through the ventricle of *Tg(myl7:LifeAct-GFP)* transgenic embryos visualising the myocardial wall in wild-type (D) and *lamb1a* mutant embryos (E-H) at 76hpf. Scale bar = 25µm. Cardiomyocytes can be seen extruding apically from the ventricular wall in *lamb1a* mutants (arrowheads E-H).

Development • Supplementary information

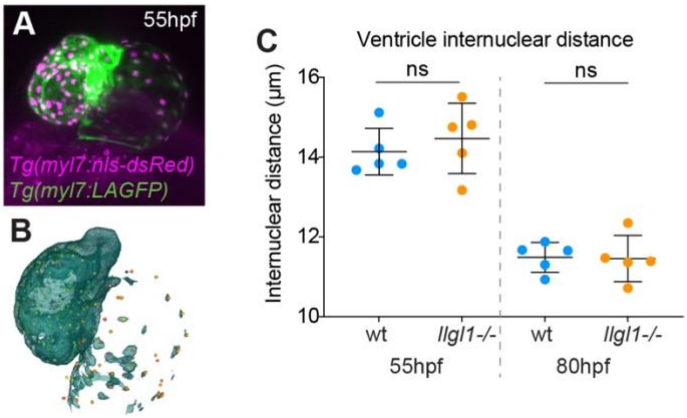

Fig. S7. Ventricular cardiomyocyte number and size is unaffected in *llgl1* mutants. A: Live lightsheet maximum intensity projection of *Tg(myl7:LifeActGFP);Tg(myl7:dsRed2)* double transgenic embryo at 55hpf, used for cell quantification, B: morphoCell reconstruction of cardiomyocyte nuclei in the whole heart, ventricular CMs in yellow, atrial CMs in orange. C: Quantification of ventricular cardiomyocyte internuclear distance as a proxy for cardiomyocyte size wild-type embryos and *llgl1* mutants at 55hpf and 80hpf (n=5 for each stage/genotype). Each point represents the average internuclear distance of the ventricular cells in an individual embryo. Cell size is comparable between wild-type embryos and *llgl1* mutants at both stages. T-test, ns = non significant.

Development • Supplementary information

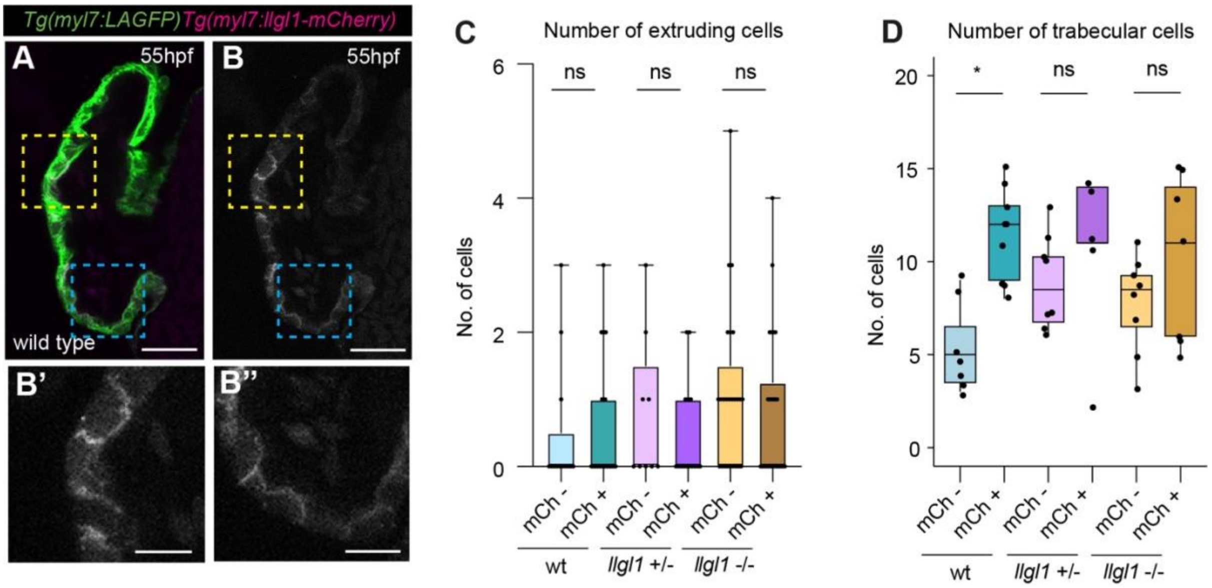

Fig. S8. Myocardial re-expression of *llgl1* causes ventricular wall defects.

A-B: Confocal z-slices of the ventricle of *Tg(myl7:LifeAct-GFP);Tg(myl7:llgl1-mCherry* transgenic embryos visualising the myocardium (green) and mCherry-tagged *llgl1* expressing myocardial cells (magenta A, B) at 55hpf. Scale bar = 25µm. B' and B'' show higher magnifications of the yellow and blue dashed boxes, respectively, shown in panels A,B, scale bar = 10µm. B,B'' Llgl1-mCherry is enriched at the basement membrane of cardiomyocytes. C: Quantification of extruding cell number in *Tg(myl7:llgl1-mCherry)*-positive wild type (n=25), *llgl1* heterozygote (n=15), and *llgl1* mutant embryos (n=26) and transgene-negative wild type (n=13), *llgl1* heterozygote (n=9), and *llgl1* mutant embryos (n=25) at 80hpf. No significant difference in the number of extruding cells is observed between *Tg(myl7:llgl1-mCherry)*-positive or -negative genotypes (Kruskal-Wallis test). D: Quantification of trabecular cell number in *Tg(myl7:llgl1-mCherry)*-positive wild type (n=9), *llgl1* heterozygote (n=6), and *llgl1* mutant embryos (n=8) and transgene-negative wild type (n=7), *llgl1* heterozygote (n=8), and *llgl1* mutant embryos (n=4) at 80hpf. Expression of *llgl1* under the *myl7* promoter causes defects in trabecular cell number in wild type embryos (Kruskal-Wallis test, * $p<0.05$, ns = non significant).

Development • Supplementary information

Table S1. Number of hearts with transplanted cells, cells targeted to myocardium, and hearts that displayed apical cell extrusion

Donor genotype to host genotype	No. embryos with transplanted cells in heart	No. hearts with cells transplanted into myocardium	No. hearts with transplanted extruding cardiomyocytes
wt to wt	5	2	0
wt to *llgl1* +/-	12	4	0
wt to *llgl1* -/-	8	4	0
llgl1 +/- to wt	12	3	1
llgl1 +/- to *llgl1* +/-	37	25	0
llgl1 +/- to *llgl1* -/-	13	5	2
llgl1 -/- to wt	11	5	0
llgl1 -/- to *llgl1* +/-	13	7	1
llgl1 -/- to *llgl1* -/-	4	3	0

Development • Supplementary information
