# Supplementary material for: Llgl1 mediates timely epicardial emergence and establishment of an apical laminin sheath around the trabeculating cardiac ventricle
Source: Development. 2024 Jun 28;151(13):dev202482. doi: 10.1242/dev.202482 (PMC11234374; doi:10.1242/dev.202482)
Supplement: Supplementary file 1 [file develop-151-202482_review_history.pdf]

Llgl1 mediates timely epicardial emergence and establishment of an apical laminin sheath around the trabeculating cardiac ventricle.

Eric JG Pollitt, Juliana Sanchez-Posada, Corinna M Snashall, Christopher J Derrick and Emily Noel

DOI: 10.1242/dev.202482

Editor: Benoit Bruneau

Review timeline

Submission to Review Commons:	16 August 2023
Submission to Journal of Cell Science:	26 October 2023
Editorial decision:	1 November 2023
First revision received:	30 April 2024
Accepted:	31 May 2024

Reviewer 1

Evidence, reproducibility and clarity

Pollitt et al. investigated the role of Llgl1 in maturation of the ventricular wall in zebrafish. They examined zebrafish heart morphology with microscopy analysis of fluorescent reporter lines crossed with their CRISPR/Cas9 llgl1 line and observed apically extruding CMs in llgl1 mutant embryos, implicating a role for llgl1 in ventricular wall integrity. Further, they examined apicobasal polarity in the ventricular wall through quantification of Crb2a distribution at the apical membrane surface, with enhanced Crb2a retention at CM junctions in llgl1 mutant embryos in comparison to WT at 72 hpf but similar levels at 80 hpf. Further analyses indicated a requirement for llgl1 for the temporal establishment of the apical laminin sheath, llgl1 is required for the timely dissemination of epicardial cells which deposit laminin to maintain the integrity of the ventricular wall. This work is written well and provides new information on heart development in zebrafish, and provides additional information on the role of the epicardium in supporting the integrity of the ventricular wall during trabeculation.

Major:

1. Although information is provided in the introduction and discussion on the role of the Llgl1 homolog in Drosophila and speculation on LLGL1 contributing to heart defects in SMS patients in the discussion, have Llgl1 homologs been examined in other vertebrate animal models during heart development or regeneration?
2. In Fig. 3I-O: The authors described the spatial dynamics of laminin in llgl1 mutants at 72 and 80m hpf. However, it is hard to say the schematic depicting of laminin for llg1 mutant in Fig. 3O reflect the real laminin staining signals in Fig. 3J' and 3L'.
3. It is mentioned that llgl1 CRISPR/Cas9 mutants are viable as adults on pg. 3 of the Results section. Have the authors examined heart morphology in these mutants in juvenile or adult fish?
4. In Fig. 4J-M', there is no Cav1 signals after wt1a MO but still laminin signals. Where these laminins come from?
5. As pan-epicardial transgenes like tcf21 reporters have been widely used, the authors should use such reporters to verify the expression of laminin gene expression in epicardial cells, and the efficacy and efficiency of depleting epicardial cells after wt1 MO injection.

© 2024. Published by The Company of Biologists under the terms of the Creative Commons Attribution License (https://creativecommons.org/licenses/by/4.0/).

Minor:

1. On page 3 of the manuscript, Fig. 1A should be included with Fig. 1B in the first sentence of paragraph 2 of the Results subsection "Llgl1 regulates ventricular wall integrity and trabeculation".
2. Fig. 2E: Is Fig. 2E from WT or llgl1 embryos? This information isn't indicated in the image panels or the figure legend. It might also be beneficial to include a similar representative image for the WT or llgl1 mutant embryo as this was used for quantification.
3. Fig. 3G: As the entirety of Fig. 3 used violet coloring to depict Laminin, it would be more consistent to change the blue coloring used to depict Laminin in panel G to the same violet coloring used for Laminin in the other panels of Fig. 3.
4. It would be beneficial to readers to briefly describe what cell type the transgenic reporters label when mentioned in the Results section to help readers unfamiliar with zebrafish.

Significance

This work is written well and provides new information on heart development in zebrafish, and provides additional information on the role of the epicardium in supporting the integrity of the ventricular wall during trabeculation.

Reviewer 2

Evidence, reproducibility and clarity

Summary: The manuscript by Pollitt et al. explores the functions of llgl1, which encodes a critical component of the basolateral domain complex, during cardiac development in zebrafish. The authors observed that llgl1 mutants exhibited compromised myocardial tissue integrity with significantly higher numbers of apically extruding cardiomyocytes. Llgl1 appears to primarily function during epicardial cell spreading on the myocardial tissue, as myocardial-specific overexpression of llgl1 did not rescue llgl1 mutant phenotypes. llgl1 mutants exhibited impaired epicardial coverage and subsequently Laminin deposition on the apical side of the cardiomyocytes. Functional linkage between Laminin/the basement membrane was identified, as extruding cardiomyocytes were also observed in mutants of two core laminin genes, lamb1a and lamc1. The epicardial defects were transmitted to myocardial tissue defects, marked by mis-localization of the apical polarity protein Crumbs2a during early heart development. Overall, the authors provide a nice study that strengthens the role of apicobasal factors in myocardial tissue morphogenesis and that sheds light on the role of epicardial-derived basement membrane in maintaining myocardial tissue integrity.

Major comments

- The authors note an interesting observation with apical and basal laminin deposition dynamics surrounding cardiomyocytes, and that Llg1 has a role in apical Laminin deposition (however, highly variable at 80 hpf as Figure 3M shows). They carry out a very nice study in which they overexpress Llgl1 tagged with mCherry in the myocardium and show that there is no rescue of the extruding cardiomyocyte defect or Laminin deposition. However, there is still a possibility that the tagged Llgl1 in the transgene Tg(myl7:Llg1-mCherry)sh679 might not be functional due to improper protein folding or interference by the mCherry tag. The authors should supplement their approach with a transplantation experiment to generate mosaic llgl1 mutant animals and assess whether llgl1 mutant cardiomyocytes extrude at a higher rate than the control. This would provide definitive evidence that Llg1l acts in a cell non- autonomous manner.
- The data in this manuscript appears to point that Llgl1 regulates Laminin deposition mainly in epicardial cells to regulate their dissemination/migration across the ventricular myocardial surface. It would be important to test this cell-autonomous function with the transplant experiment (above point) and examine whether llgl1 mutant epicardial cells fail to migrate and deposit Laminin. It might be possible to perform a rescue experiment through overexpression of Llgl1 in epicardial cells (if possible, there is a tcf21:Gal4 line available).

© 2024. Published by The Company of Biologists under the terms of the Creative Commons Attribution License (https://creativecommons.org/licenses/by/4.0/).

- In the Discussion, the authors propose that Llgl1 acts in two ways: Laminin deposition in epicardial cells that suppress cell extrusion and polarity regulation in cardiomyocytes to promote trabeculation. It would be important to test the second hypothesis on trabeculation and polarity regulation by using the myocardial-specific overexpression/rescue of Llgl1 in llgl1 mutants, and then quantifying the trabeculating cardiomyocytes and analyze Crb2a localization. This experiment can distinguish whether this trabeculation phenotype is rescued independently of the apical Laminin deposition that has been included in Figure S5.
- The potential mis-localization of Crb2a in the llgl1 mutants is interesting, but this effect appears to be quite mild, and as the authors note, resolve by 80 hpf. Considering the role of Lgl in Drosophila in shifting Crb complex localization during early epithelial morphogenesis, it would be worth performing the analysis at earlier timepoints (between 55 and 72 hpf) to determine whether Llgl1 is indeed important for the progressive apical relocalization of Crb2a.
OPTIONAL: It might be worth testing other antibodies that could mark the apical (particularly aPKC which is known to phosphorylate and regulate the Crb complex) and basolateral domains (Par1, Dlg) of the cardiomyocytes to definitively conclude that the epithelial integrity of the cells is affected. Although there are no reports of working antibodies marking the basal domain in zebrafish, there is at least a Tg(myl7:MARCK3A-RFP) line published (Jimenez- Amilburu et al. (2016)) - which the authors can inject to examine the localization in mosaic hearts.
- Have the authors quantified the numbers of total cardiomyocytes in llgl1 mutants to correlate how many cells are lost as a consequence of extrusion? What is the physiological impact of this extrusion (ejection fraction, total cardiac volumes, sarcomere organization)?
- The lamb1a and lamc1 mutant phenotypes were nicely analyzed. However, there is basement membrane deposition on both the apical and basal sides of the cardiomyocytes. Therefore, it is unclear whether the cardiomyocyte extrusion is completely caused by loss of apical basement membrane, or whether the loss of basal basement membrane could compromise the myocardial tissue integrity. The authors should clarify this conclusion in the text.

Minor comments

- The authors note that Llgl1-mCherry in the Tg(myl7:Llg1-mCherry)sh679 line localizes to the basolateral domain of the cardiomyocytes, which is valuable confirmation that Llgl1 protein is spatially restricted. However, only 1 timepoint (55 hpf) is noted. It would be important to perform Llgl1 localization across different developmental timepoints (at least until 80 hpf) to examine the dynamics of this protein during trabeculation and apical extrusion, and potentially correlate it with Crb2a localization for a better understanding of the apicobasal machinery in cardiomyocytes.
- The phenotypes of llgl1 mutants described here differ compared to the previous study by Flinn et al. (2020). In particular, whereas the mutants generated in this study have only mild pericardial edema and are adult viable, approximately one third of llgl1mw3 (Flinn et al. (2020)) died at 6 dpf. Is this caused by the different natures of the mutations in the llgl1 gene? Is there a possibility that the llgl1sh598 is a hypomorphic allele since the targeted deletion is in a more downstream sequence (in exon 2) compared to the llgl1mw3 (deletion in exon 1) allele?
Suggested experiment: qPCR of regions downstream of the deletion to make sure that the transcript is absent/reduced in the llgl1sh598 mutants. Alternatively, immunostaining or Western blot would be an even better option to ensure there is no Llgl1 protein production - there is an anti-Llgl1 antibody available that works for Western blots in zebrafish (Clark et al. (2012)).
- Closeups needed for Figure 3I-L' - difficult to assess mis-localization or differences in Laminin staining. Contrary to the quantification or conclusion, the Laminin staining appears stronger in llgl1 mutants compared to wild types in Figure 3I' and J'.
OPTIONAL: Gentile et al. (2021) found that reducing heartbeat led to decreased cardiomyocyte extrusion in snai1b mutants. The authors could look into the contribution of mechanical pressure through contraction in the apical cardiomyocyte extrusion, and test whether reducing contraction (tnnt2 morpholino, chemical treatments) partly rescues the llgl1 mutant phenotypes.

Significance

As someone with expertise in cardiac development and cellular behaviours, I find this study provides strong and convincing quantitative data on the role of Llgl1 in suppressing cardiomyocyte extrusion and promoting epicardial dissemination on the ventricular surface. The genetic

© 2024. Published by The Company of Biologists under the terms of the Creative Commons Attribution License (https://creativecommons.org/licenses/by/4.0/).

experiments, including mutant analysis and myocardial-specific rescue, were carefully performed in a region-specific manner, which provides much insight into the non- uniformity of myocardial tissue integrity. The generation of Tg(myl7:llgl1-mCherry) line is also a valuable tool for researchers in the field interested in understanding apicobasal polarity and cardiomyocyte development and regeneration.

A limitation of the study is the unclear link between epithelial polarity and basement membrane deposition, and how they synchronize to regulate cardiomyocyte integrity. The llgl1 mutant phenotype in increasing cardiomyocyte apical extrusion and Crb2 localization is interesting; however, the authors note that this appears to be a phenotype induced by epicardial defects. Epicardial cells are not known to exhibit apicobasal polarity and are fibroblastic by nature. Thus, the cellular mechanisms by which Llg1 regulates epicardial cell morphology or behaviours, and how it functions to regulate polarity in cardiomyocytes are not clearly defined in this work. In addition, clarification of the cell autonomous functions of Llgl1 in epicardial cells and/or cardiomyocytes would strengthen the findings.

Overall, the findings of this study would be of interest to cell and developmental biologists in the fields of epithelial polarity, cardiac morphogenesis, and extracellular matrix function. It provides nice conceptual advance in further elucidating the mechanisms that underlie myocardial tissue integrity and epicardial-myocardial interactions.

Reviewer 3

Evidence, reproducibility and clarity

This manuscript from Pollitt and colleagues analyzes the role of lethal(2) giant larvae homolog 1 (llgl1) in cardiac development in zebrafish. Llgl1 has been previously involved in regulating epithelial polarity, which raises the possibility that this gene might play a role during the trabeculation of the zebrafish heart. To examine the role of llgl1 in this phenomenon, the authors generated a new loss of function mutant using CRISPR/Cas9.
Animals lacking llgl1 initially exhibited abnormal cardiac development, manifested by defects in cardiac looping and pericardial edema. This phenotype, however, was transient. A detailed analysis of the developing heart showed, albeit with significant variability, defects in trabeculation and an interesting cardiomyocyte extrusion phenotype, described before in mutants that lack epicardium. During their analysis, the authors discovered a switch in the localization of the extracellular matrix protein laminin from the luminal to the apical side of the cardiomyocytes that temporally correlates with the process of trabeculation. The accumulation of laminin in the epicardial side was affected in llgl1 mutants, which also showed a defect in epicardial development. Coincidentally, the epicardial cells appear to be the primary source of laminin. This work suggests that llgl1 acts in epicardial cells to maintain ventricular wall integrity during heart development.

Major Comments:

1. Major - the authors describe that llgl1 mutants exhibit transient cardiac edema at 3 dpf, which is resolved by 5 dpf, and claim that the mutants are viable. This statement needs to be better supported - What is the proportion of mutants that survive to adulthood? The embryonic phenotypes are pretty variable - are the mutants that survive the ones with a less severe phenotype? Is there a gross defect in the adult heart of these animals?
2. Major - Many of the phenotypes described here -most importantly, the defects on epicardial development- could result from hemodynamic defects in llgl1 mutants. The authors claim that function is unaffected in these animals, but this has only been addressed by measuring heartbeat. The observation that the cardiac function in these animals is normal would conflict with a previous description (PMID: 32843528) that demonstrates that llgl1 mutant animals show significant hemodynamic defects, which would cause epicardial defects. Thus, this aspect of the work needs to be better addressed.
3. The phenotypes related to forming multiple layers in the heart (Fig. 1) could be more convincing. In some figures, the authors use a reporter that labels the myocardial cell membrane, but in Figure 1 this is not used. Showing a myocardial membrane marker (for

© 2024. Published by The Company of Biologists under the terms of the Creative Commons Attribution License (https://creativecommons.org/licenses/by/4.0/).

example, the antibody Alcama, Zn-8) would significantly strengthen this observation.
4. The analysis of Crumbs redistribution (Fig. 2) is quite interesting. Still, given that the authors have a transgenic model to rescue llgl1 expression in cardiomyocytes, they could move from correlative evidence to experimental demonstration of the role of llgl1 in Crumbs localization.
5. (Optional) There is laminin in the luminal side of the heart before there is any epicardial invasion. What is the source of this laminin? The techniques the authors have used (i.e., chromogenic ISH) are fine, but a more detailed analysis using fluorescent ISH (i.e., RNAScope) would be much more definitive.
6. How llgl1 relates to epicardial biology is left entirely unexplored in this work. Do proepicardial cells show any defect in cell polarization related to llgl1 absence?

Significance

General Assessment. Overall, this is an interesting manuscript put together with rigor. The strongest aspect of this work is the discovery of a switch in the localization of laminin in the developing heart and the potential implications of this process in regulating correct trabeculation versus cardiomyocyte extrusion. Although the text itself is very well written, with clear statements of the hypotheses and the findings that led the authors to each experiment, I found myself wondering what the unifying theme and central message of the manuscript is and whether this has been appropriately supported with experimental data. Specifically, although the authors included a very detailed analysis of the myocardium, their results (including the last supplementary figure) suggest that these phenotypes might be secondary to a defect in epicardial development. Still, it is entirely unclear how the loss of llgl1 would affect epicardial development.

Author response to reviewers' comments

Manuscript number: RC-2023-02134
Corresponding author(s): Emily Noël

[The “revision plan” should delineate the revisions that authors intend to carry out in response to the points raised by the referees. It also provides the authors with the opportunity to explain their view of the paper and of the referee reports.

1. General Statements [optional]

In this paper we describe the new finding that the epicardial deposits the extracellular matrix component laminin onto the apical ventricular surface during cardiac development. We identify a novel role for the apicobasal polarity protein Llgl1in timely emergence of the epicardium and deposition of this apical laminin, alongside a requirement for Llgl1 in maintaining integrity of the ventricular wall at the onset of trabeculation.
We thank the reviewers for their very positive appraisal of our manuscript, and for their helpful suggestions for useful revisions. In particular we would like to highlight the broad interest they feel this manuscript holds, not only contributing conceptual advances to our understanding of multiple aspects of cardiac development, but also to cell and developmental biologists working in epithelial polarity and extracellular matrix function. We also note their positive appraisal of the rigor of the study and quality of the manuscript.

2. Description of the planned revisions

Reviewer 1
1a) It is mentioned that llgl1 CRISPR/Cas9 mutants are viable as adults on pg. 3 of the Results section. Have the authors examined heart morphology in these mutants in juvenile or adult fish?
* We have some historical data on adult *llgl1* mutant survival that we plan to include in the study.

Reviewer 2
2a) The authors note an interesting observation with apical and basal laminin deposition dynamics surrounding cardiomyocytes, and that Llg1 has a role in apical Laminin deposition (however, highly variable at 80 hpf as Figure 3M shows). They carry out a very nice study in which they overexpress Llgl1 tagged with mCherry in the myocardium and show that there is no rescue of the extruding

© 2024. Published by The Company of Biologists under the terms of the Creative Commons Attribution License (https://creativecommons.org/licenses/by/4.0/).

cardiomyocyte defect or Laminin deposition. However, there is still a possibility that the tagged Llgl1 in the transgene Tg(myl7:Llg1-mCherry)sh679 might not be functional due to improper protein folding or interference by the mCherry tag. The authors should supplement their approach with a transplantation experiment to generate mosaic llgl1 mutant animals and assess whether llgl1 mutant cardiomyocytes extrude at a higher rate than the control. This would provide definitive evidence that Llg1l acts in a cell non-autonomous manner.
* We agree with the reviewer, and propose to perform transplant experiments, transplanting cells from *llgl1* mutants into wild type siblings, and quantify cell extrusion to determine whether *llgl1* mutant cells are extruded more frequently than wild type.

2b) The data in this manuscript appears to point that Llgl1 regulates Laminin deposition mainly in epicardial cells to regulate their dissemination/migration across the ventricular myocardial surface. It would be important to test this cell-autonomous function with the transplant experiment (above point) and examine whether llgl1 mutant epicardial cells fail to migrate and deposit Laminin. It might be possible to perform a rescue experiment through overexpression of Llgl1 in epicardial cells (if possible, there is a tcf21:Gal4 line available).
* Similar to above, we propose to perform transplant experiments, transplanting cells from *llgl1* mutants or wild type siblings into wild type siblings or *llgl1* mutants, respectively, and in this instance quantify contribution of transplanted cells to epicardial coverage.

2c) In the Discussion, the authors propose that Llgl1 acts in two ways: Laminin deposition in epicardial cells that suppress cell extrusion and polarity regulation in cardiomyocytes to promote trabeculation. It would be important to test the second hypothesis on trabeculation and polarity regulation by using the myocardial-specific overexpression/rescue of Llgl1 in llgl1 mutants, and then quantifying the trabeculating cardiomyocytes and analyze Crb2a localization. This experiment can distinguish whether this trabeculation phenotype is rescued independently of the apical Laminin deposition that has been included in Figure S5.
* To help address the second part of our hypothesis laid out in the discussion, we propose to quantify trabecular organisation and Crb2a localisation in *llgl1* mutants either carrying the *myl7:llgl1-mCherry* construct, or mCherry-negative controls.

2d) The potential mis-localization of Crb2a in the llgl1 mutants is interesting, but this effect appears to be quite mild, and as the authors note, resolve by 80 hpf. Considering the role of Lgl in Drosophila in shifting Crb complex localization during early epithelial morphogenesis, it would be worth performing the analysis at earlier timepoints (between 55 and 72 hpf) to determine whether Llgl1 is indeed important for the progressive apical relocalization of Crb2a.
* We will expand our description of this in the mutants by performing analysis of Crb2a at earlier timepoints in the *llgl1* mutant (55hpf and 60hpf)

2e) OPTIONAL: It might be worth testing other antibodies that could mark the apical (particularly aPKC which is known to phosphorylate and regulate the Crb complex) and basolateral domains (Par1, Dlg) of the cardiomyocytes to definitively conclude that the epithelial integrity of the cells is affected. Although there are no reports of working antibodies marking the basal domain in zebrafish, there is at least a Tg(myl7:MARCK3A-RFP) line published (Jimenez-Amilburu et al. (2016)) - which the authors can inject to examine the localization in mosaic hearts.
* We plan to assess localisation of aPKC (see section 4 for response to other suggested polarity protein analyses).

2f) Have the authors quantified the numbers of total cardiomyocytes in llgl1 mutants to correlate how many cells are lost as a consequence of extrusion? What is the physiological impact of this extrusion (ejection fraction, total cardiac volumes, sarcomere organization)?
* We have some of this data already which we will include in the manuscript (cell number, myocardial volume). We agree that the analysis of cardiac function could be more extensive, and we will perform more detailed analysis of cardiac function, including e.g. ejection fraction. Sarcomere organisation has been previously described in *llgl1* mutants by Flinn et al, 2020, so we do not plan to replicate this data.

2g) The lamb1a and lamc1 mutant phenotypes were nicely analyzed. However, there is basement membrane deposition on both the apical and basal sides of the cardiomyocytes. Therefore, it is unclear whether the cardiomyocyte extrusion is completely caused by loss of apical basement

© 2024. Published by The Company of Biologists under the terms of the Creative Commons Attribution License (https://creativecommons.org/licenses/by/4.0/).

membrane, or whether the loss of basal basement membrane could compromise the myocardial tissue integrity. The authors should clarify this conclusion in the text.
* We will address this further in the text, but will also include 55hpf Laminin staining data for *llgl1* mutants to reinforce our message.

2h) The authors note that Llgl1-mCherry in the Tg(myl7:Llg1-mCherry)sh679 line localizes to the basolateral domain of the cardiomyocytes, which is valuable confirmation that Llgl1 protein is spatially restricted. However, only 1 timepoint (55 hpf) is noted. It would be important to perform Llgl1 localization across different developmental timepoints (at least until 80 hpf) to examine the dynamics of this protein during trabeculation and apical extrusion, and potentially correlate it with Crb2a localization for a better understanding of the apicobasal machinery in cardiomyocytes.
* We already have some of this data and will include extra timepoints in a revised version of the manuscript

2i) The phenotypes of llgl1 mutants described here differ compared to the previous study by Flinn et al. (2020). In particular, whereas the mutants generated in this study have only mild pericardial edema and are adult viable, approximately one third of llgl1mw3 (Flinn et al. (2020)) died at 6 dpf. Is this caused by the different natures of the mutations in the llgl1 gene? Is there a possibility that the llgl1sh598 is a hypomorphic allele since the targeted deletion is in a more downstream sequence (in exon 2) compared to the llgl1mw3 (deletion in exon 1) allele?
* We thank the reviewer for noticing these subtle differences between the two *llgl1* mutants. Indeed, while we occasionally see *llgl1*^{sh598} mutants with the severe phenotype described by Flinn et al, this is a small minority which we did not quantify. Our mutation is indeed slightly further downstream than that described by Flinn et al, however we believe that this will have a neglible effect on Llgl1 function. Our *llgl1*^{sh589} mutation results in truncation shortly into the WD40 domain, and importantly completely lacks the Lgl-like domain, which is responsible for the specific function of Llgl1 likely through its ability to interact with SNAREs to regulate cargo delivery to membranes (Gangar et al, Current Biology 2005).
Interestingly, Flinn et al report no increased phenotypic severity in their maternal-zygotic *llgl1* mutants when compared to zygotic mutants. Conversely, we often observed very severe phenotypes in *MZ llgl1*^{sh589} mutants, including failure of embryos during blastula stages, apparently through poor blastula integrity. We did not include this information in the manuscript due to space constraints. However, we argue that together these differences between the two alleles may not be due to hypomorphism of our *llgl1*^{sh589} allele, but rather differences in genetic background that may amplify specific phenotypes. We plan to include a short sentence summarising the above in combination with planned experiments described below to address the reviewer's next comment.

2j) Suggested experiment: qPCR of regions downstream of the deletion to make sure that the transcript is absent/reduced in the llgl1sh598 mutants. Alternatively, immunostaining or Western blot would be an even better option to ensure there is no Llgl1 protein production - there is an anti-Llgl1 antibody available that works for Western blots in zebrafish (Clark et al. (2012)).
* We plan to analyse *llgl1* expression in *llgl1* mutants using qPCR.

Reviewer 3
3a) Major - the authors describe that llgl1 mutants exhibit transient cardiac edema at 3 dpf, which is resolved by 5 dpf, and claim that the mutants are viable. This statement needs to be better supported - What is the proportion of mutants that survive to adulthood? The embryonic phenotypes are pretty variable - are the mutants that survive the ones with a less severe phenotype? Is there a gross defect in the adult heart of these animals?
* In line with comments from Reviewers 1 and 2 above, we will include a description of the data we have from adult animals (historical data, not generation of new animals).

3b) Major - Many of the phenotypes described here -most importantly, the defects on epicardial development- could result from hemodynamic defects in llgl1 mutants. The authors claim that function is unaffected in these animals, but this has only been addressed by measuring heartbeat. The observation that the cardiac function in these animals is normal would conflict with a previous description (PMID: 32843528) that demonstrates that llgl1 mutant animals show significant hemodynamic defects, which would cause epicardial defects. Thus, this aspect of the work needs to be better addressed.

© 2024. Published by The Company of Biologists under the terms of the Creative Commons Attribution License (https://creativecommons.org/licenses/by/4.0/).

* In line with our comments to point 2f) from Reviewer 2, we will perform a more in-depth functional analysis on *llgl1* mutant larvae.

3c) The phenotypes related to forming multiple layers in the heart (Fig. 1) could be more convincing. In some figures, the authors use a reporter that labels the myocardial cell membrane, but in Figure 1 this is not used. Showing a myocardial membrane marker (for example, the antibody Alcama, Zn-8) would significantly strengthen this observation.
* We will describe trabecular phenotypes in more detail using the suggested antibody to highlight membranes.

3d) The analysis of Crumbs redistribution (Fig. 2) is quite interesting. Still, given that the authors have a transgenic model to rescue llgl1 expression in cardiomyocytes, they could move from correlative evidence to experimental demonstration of the role of llgl1 in Crumbs localization.
* Similar to our response to comment 2c) from Reviewer 2, we plan to address this

3. Description of the revisions that have already been incorporated in the transferred manuscript

Reviewer 1:
Although information is provided in the introduction and discussion on the role of the Llgl1 homolog in Drosophila and speculation on LLGL1 contributing to heart defects in SMS patients in the discussion, have Llgl1 homologs been examined in other vertebrate animal models during heart development or regeneration?
* With the exception of the Flinn et al paper, we find no published studies assessing the role of Llgl1 in heart development or regeneration in other vertebrates, and have updated the introduction to highlight this fact:
'Zebrafish have two *Lgl* homologues, *llgl1* and *llgl2*, and *llgl1* has previously been shown to be required for early stages of heart morphogenesis (Flinn et al. 2020). However, although *Llgl1* expression has also been reported in the developing mouse heart and both adult mouse and human hearts (Uhlén et al. 2015; Klezovitch et al. 2004), whether *llgl1* plays a role in ventricular wall development has not been examined.'

In Fig. 4J-M', there is no Cav1 signals after wt1a MO but still laminin signals. Where these laminins come from?
* The residual laminin staining observed in *wt1a* morphants is located at the basal surface of cardiomyocytes (while the apical laminin signal is lost, in line with the epicardial deposition of laminin at the apical ventricle surface). This basal laminin is likely deposited earlier during heart tube development by either the myocardium, endocardium or both, and thus unaffected by later formation of the epicardium. We reason this since a) it is present at the basal cardiomyocyte surface at 55hpf (see Fig 2); b) we have previously identified both myocardial and endocardial expression of *laminin* subunits at 26hpf and 55hpf (Derrick et al, Development, 2021); c) sc-RNA-seq analysis of hearts at 48hpf demonstrates that laminin subunits, e.g. *lamc1* are expressed in myocardial and endocardial cells (Nahia et al, bioRxiv, 2023), also in line with our previous ISH analysis. We have included a sentence to reflect this in the results section:
`Conversely, *wt1a* morphants retain deposition of laminin at the basal CM surface, likely from earlier expression and deposition of laminin by either myocardial or endocardial cells (Derrick et al. 2021; Nahia et al. 2023), which is unaffected by later epicardial development.`

On page 3 of the manuscript, Fig. 1A should be included with Fig. 1B in the first sentence of paragraph 2 of the Results subsection "Llgl1 regulates ventricular wall integrity and trabeculation".
* Amended

It would be beneficial to readers to briefly describe what cell type the transgenic reporters label when mentioned in the Results section to help readers unfamiliar with zebrafish.
* We have updated the text to read:
`We further analysed heart morphology using live lightsheet microscopy of *Tg(myl7:LifeActGFP);Tg(fli1a:AC-TagRFP)* double transgenic wild-type and *llgl1* mutant embryos, allowing visualisation of myocardium (green) and endocardium (magenta) respectively. Comparative analysis of overall heart morphology between 55hpf and 120hpf when looping morphogenesis is complete, revealing that *llgl1* mutants continue to exhibit defects in heart morphogenesis (Fig S1S-X).`

© 2024. Published by The Company of Biologists under the terms of the Creative Commons Attribution License (https://creativecommons.org/licenses/by/4.0/).

Reviewer 3
(Optional) There is laminin in the luminal side of the heart before there is any epicardial invasion. What is the source of this laminin? The techniques the authors have used (i.e., chromogenic ISH) are fine, but a more detailed analysis using fluorescent ISH (i.e., RNAScope) would be much more definitive.
* This is related to our response to Reviewer 1 (above) – where we have included the following text included in manuscript: `Conversely, *wt1a* morphants retain deposition of laminin at the basal CM surface, likely from earlier expression and deposition of laminin by either myocardial or endocardial cells (Derrick et al. 2021; Nahia et al. 2023), which is unaffected by later epicardial development.` We hope this clarifies our proposed origins for the earlier laminin deposition.

4. Description of analyses that authors prefer not to carry out

Reviewer 1:
5. As pan-epicardial transgenes like tcf21 reporters have been widely used, the authors should use such reporters to verify the expression of laminin gene expression in epicardial cells, and the efficacy and efficiency of depleting epicardial cells after wt1 MO injection.
* Several studies have demonstrated that the epicardium is not a heterogeneous population – for example, *tcf21* is not expressed in all epicardial cells and thus not a pan-epicardial reporter (Plavicki et al, BMC Dev Biol, 2014, Weinberger et al, Dev Cell, 2020) – the suggested analysis would not necessarily be conclusive, and more detailed study would require acquisition of three new transgenic lines. Furthermore, we believe the evidence we present in the paper supports our claim: 1) We show expression of two laminin subunits in a thin mesothelial layer directly adjacent to the myocardium, specifically in the location of the epicardium; 2) sc-RNA seq analyses have also identified laminin expression in epicardial cells at 72hpf (where *lamc1a* is identified as a marker of the epicardium); 3) We demonstrate 100% efficacy of our *wt1a* knockdown as assayed by Cav1 expression, an established epicardial marker (Grivas et al, 2020, Marques et al, 2022) which in sc-RNA seq data is expressed at high levels broadly in the epicardial cell population (Nahia et al, 2023), representing a good assay for presence of epicardium. However, we propose to perform ISH analysis of laminin subunit expression in *wt1a* MO to investigate whether the mesothelial laminin-expressing layer we observe adjacent to the myocardium is absent upon loss of *wt1a*.

Reviewer 2:
- The data in this manuscript appears to point that Llgl1 regulates Laminin deposition mainly in epicardial cells to regulate their dissemination/migration across the ventricular myocardial surface. It would be important to test this cell-autonomous function with the transplant experiment (above point) and examine whether llgl1 mutant epicardial cells fail to migrate and deposit Laminin. It might be possible to perform a rescue experiment through overexpression of Llgl1 in epicardial cells (if possible, there is a tcf21:Gal4 line available).
* We do not propose to perform this experiment using a tcf21:Gal4 line, as this would likely require at least 6 months to either import and quarantine, or generate the necessary stable lines. Furthermore, as mentioned above, *tcf21* is not a pan-epicardial marker, and the extent and timing of the Gal4:UAS system may make this challenging to determine whether *llgl1* has been expressed early or broadly enough. We will instead attempt transplantation experiments.

OPTIONAL: It might be worth testing other antibodies that could mark the apical (particularly aPKC which is known to phosphorylate and regulate the Crb complex) and basolateral domains (Par1, Dlg) of the cardiomyocytes to definitively conclude that the epithelial integrity of the cells is affected. Although there are no reports of working antibodies marking the basal domain in zebrafish, there is at least a Tg(myl7:MARCK3A-RFP) line published (Jimenez-Amilburu et al. (2016)) - which the authors can inject to examine the localization in mosaic hearts.
* We will assess localisation of aPKC, but we do not plan to analyse the other components. Analysis of basolateral domains (Par1, Dlg, Mark3a-RGP), will not necessarily assess epithelial integrity, as suggested, but rather apicobasal polarity – which we already assess using Crb2a, and additionally plan to assess aPKC to accompany the Crb2a analysis. Since the reviewer suggests this as an optional experiment we prioritise their other suggested experiments that we think more directly address the main messages of the manuscript.

- OPTIONAL: Gentile et al. (2021) found that reducing heartbeat led to decreased cardiomyocyte extrusion in snai1b mutants. The authors could look into the contribution of mechanical pressure

© 2024. Published by The Company of Biologists under the terms of the Creative Commons Attribution License (https://creativecommons.org/licenses/by/4.0/).

through contraction in the apical cardiomyocyte extrusion, and test whether reducing contraction (tnnt2 morpholino, chemical treatments) partly rescues the llgl1 mutant phenotypes.
* The relationship between cardiac function and myocardial wall integrity appears to be complex. The paper referred to by the reviewer indeed finds that reduction in heartbeat leads to decreased CM extrusion upon loss of the EMT-factor Snai1b. Previous studies have also found that endothelial flow-responsive genes *klf2a/b* are required to maintain myocardial ventricular wall integrity at later stages in a contractility-dependent manner (Rasouli et al, 2018). However, contractility is also required early for pro-epicardial emergence, but plays a lesser role in expansion of the epicardial layer on the myocardial surface (Peralta, 2013). Unpicking the relationship between the forces induced by mechanical contraction of the ventricular wall, contractility-based induction of e.g *klf2* expression, and the impact of contractile forces on proepicardial development or epicardial expansion will be complex. We therefore think the proposed experiment will be difficult to interpret whatever the outcome, and argue that dissecting this relationship is beyond the scope of revisions for this paper.

Reviewer 3
(6) How llgl1 relates to epicardial biology is left entirely unexplored in this work. Do proepicardial cells show any defect in cell polarization related to llgl1 absence?
* We agree with the reviewer that we do not delve into the mechanisms underlying regulation of epicardial development by *llgl1*, and that this is an interesting question. Our scope for this manuscript was to understand the mechanisms by which *llgl1* regulates integrity of the ventricular wall, and feel that uncovering the molecular mechanisms by which *llgl1* regulates timely epicardial emergence is a larger question that would require substantial investigation (for example, if and when *llgl1* PE cells do exhibit apicobasal defects, how this impacts timing of cluster release etc). We think these are important questions that would be better answered in detail in a separate manuscript.

Original submission

<u>First decision letter</u>

MS ID#: DEVELOP/2023/202482

MS TITLE: Llgl1 mediates timely epicardial emergence and establishment of an apical laminin sheath around the trabeculating cardiac ventricle.

AUTHORS: Eric JG Pollitt, Christopher J Derrick, Juliana Sanchez-Posada, and Emily Noel

I have now received all the referees' reports and your revision plan on the above manuscript, and have reached a decision.

As you noted, the referees express considerable interest in your work, but have some significant criticisms and recommend a substantial revision of your manuscript before we can consider publication. If you are able to revise the manuscript along the lines suggested and according to your revision plan, which may involve further experiments, I will be happy receive a revised version of the manuscript. Your revised paper will be re-reviewed by one or more of the original referees, and acceptance of your manuscript will depend on your addressing satisfactorily the reviewers' major concerns. Please also note that Development will normally permit only one round of major revision. If it would be helpful, you are welcome to contact us to discuss your revision in greater detail. Please send us a point-by-point response indicating your plans for addressing the referees' comments, and we will look over this and provide further guidance.

Please attend to all of the reviewers' comments and ensure that you clearly highlight all changes made in the revised manuscript. Please avoid using 'Tracked changes' in Word files as these are lost in PDF conversion. I should be grateful if you would also provide a point-by-point response detailing

© 2024. Published by The Company of Biologists under the terms of the Creative Commons Attribution License (https://creativecommons.org/licenses/by/4.0/).

how you have dealt with the points raised by the reviewers in the 'Response to Reviewers' box. If you do not agree with any of their criticisms or suggestions please explain clearly why this is so.

First revision

Author response to reviewers' comments

We thank all reviewers for their positive appraisals of our manuscript, and helpful comments to improve the strength of our study. We have addressed comments with extra experimental data to support our findings where possible or appropriate, and/or clarified statements in the text. We hope these revisions address the reviewer's comments satisfactorily, and a detailed rebuttal to each point can be found below.

Reviewer #1

Major:

1. Although information is provided in the introduction and discussion on the role of the Llgl1 homolog in Drosophila and speculation on LLGL1 contributing to heart defects in SMS patients in the discussion, have Llgl1 homologs been examined in other vertebrate animal models during heart development or regeneration?

* With the exception of the Flinn et al paper, we find no published studies assessing the role of Llgl1 in heart development or regeneration in other vertebrates, and have updated the introduction to hightlight this fact:

'Zebrafish have two *Lgl* homologues, *llgl1* and *llgl2*, and *llgl1* has previously been shown to be required for early stages of heart morphogenesis (Flinn et al. 2020). However, although *Llgl1* expression has also been reported in the developing mouse heart and both adult mouse and human hearts (Uhlén et al. 2015; Klezovitch et al. 2004), whether *llgl1* plays a role in ventricular wall development has not been examined.'

2. In Fig. 3I-O: The authors described the spatial dynamics of laminin in llgl1 mutants at 72 and 80m hpf. However, it is hard to say the schematic depicting of laminin for llg1 mutant in Fig. 3O reflect the real laminin staining signals in Fig. 3J' and 3L'.

* We have now included magnifications for these panels (now moved to Figure 4), which we hope allow a better appreciation of Laminin localisation in the *llgl1* mutants

3. It is mentioned that llgl1 CRISPR/Cas9 mutants are viable as adults on pg. 3 of the Results section. Have the authors examined heart morphology in these mutants in juvenile or adult fish?

* We had some previous data from analysis of *llgl1* adults that we have now included in the manuscript with the following text:

'Despite the fact that *llgl1* mutants have morphological heart defects, they are adult viable. We observed no overt morphological abnormalities in adult *llgl1* mutants (Fig S3A-B), and found adult mutants at approximately Mendelian ratios in a colony grown from *llgl1* heterozygous incross embryos (wild type n=8, *llgl1+/-* n=11, *llgl1-/-* n=6). Since *llgl1* mutants exhibit variable heart morphology at 55hpf, we performed lightsheet imaging of *llgl1* mutant embryos at 120hpf, separated larvae into mild and severe cardiac phenotypes, and raised each category to adulthood. Adults raised from *llgl1* mutants with either mild or severe larval heart phenotypes had comparable morphology and behaviour. Swim tunnel analysis of *llgl1* mutants exercise tolerance revealed no deficits compared to wild type siblings, suggesting cardiovascular performance was not compromised (Fig S3C), and dissected *llgl1* mutant adult hearts appeared grossly normal. However, *llgl1* mutant fish responded poorly to anaesthesia, including gill bleeding (n=3/4), delayed recovery (n=3/4), and death (n=2/4), phenotypes which were never observed in wild type or heterozygous animals (n=6).'

4. In Fig. 4J-M', there is no Cav1 signals after wt1a MO but still laminin signals. Where these laminins come from?

* The residual laminin staining observed in *wt1a* morphants is located at the basal surface of cardiomyocytes (while the apical laminin signal is lost, in line with the epicardial deposition of laminin at the apical ventricle surface). This basal laminin is likely deposited earlier during heart tube development by either the myocardium, endocardium or both, and thus unaffected by later

© 2024. Published by The Company of Biologists under the terms of the Creative Commons Attribution License (https://creativecommons.org/licenses/by/4.0/).

formation of the epicardium. We reason this since a) it is present at the basal cardiomyocyte surface at 55hpf (see Fig 3); b) we have previously identified both myocardial and endocardial expression of *laminin* subunits at 26hpf and 55hpf (Derrick et al, Development, 2021); c) sc-RNA-seq analysis of hearts at 48hpf demonstrates that laminin subunits, e.g. *lamc1* are expressed in myocardial and endocardial cells (Nahia et al, bioRxiv, 2023), also in line with our previous ISH analysis. We have included a sentence to reflect this in the results section:
`Conversely, *wt1a* morphants retain deposition of laminin at the basal CM surface, likely from earlier expression and deposition of laminin by either myocardial or endocardial cells (Derrick et al. 2021; Nahia et al. 2023), which is unaffected by later epicardial development.`.

5. As pan-epicardial transgenes like tcf21 reporters have been widely used, the authors should use such reporters to verify the expression of laminin gene expression in epicardial cells, and the efficacy and efficiency of depleting epicardial cells after wt1 MO injection.
* Several studies have demonstrated that the epicardium is not a heterogeneous population – for example, *tcf21* is not expressed in all epicardial cells and thus not a pan-epicardial reporter (Plavicki et al, BMC Dev Biol, 2014, Weinberger et al, Dev Cell, 2020), thus that analysis would not necessarily be conclusive. Furthermore, we believe the evidence we present in the paper supports our claim: 1) We show expression of two laminin subunits in a thin mesothelial layer directly adjacent to the myocardium, specifically in the location of the epicardium; 2) sc-RNA seq analyses have also identified laminin expression in epicardial cells at 72hpf (and *lamc1* is identified as a marker of the epicardium); 3) We demonstrate 100% efficacy of our *wt1a* knockdown as assayed by Cav1 expression, an established epicardial marker which in sc-RNA seq data is broadly expressed at high levels in epicardial cells (Nahia et al, 2023), representing a good assay for presence of epicardium. We now included ISH analysis of *lamb1a* and *lamc1a* mRNA expression in control MO and wt1a MO-injected embryos into Figure 5 to further strengthen our data, and demonstrate that in the absence of epicardial cells, *lamb1a* and *lamc1* expression around the ventricle is completely lost.

Minor:
1. On page 3 of the manuscript, Fig. 1A should be included with Fig. 1B in the first sentence of paragraph 2 of the Results subsection "Llgl1 regulates ventricular wall integrity and trabeculation".
* We have amended this in the manuscript.

2. Fig. 2E: Is Fig. 2E from WT or llgl1 embryos? This information isn't indicated in the image panels or the figure legend. It might also be beneficial to include a similar representative image for the WT or llgl1 mutant embryo as this was used for quantification.
* We have updated the figure, using higher magnifications of the example panels already in the figure for both wild type and mutant at 72hpf.

3. Fig. 3G: As the entirety of Fig. 3 used violet coloring to depict Laminin, it would be more consistent to change the blue coloring used to depict Laminin in panel G to the same violet coloring used for Laminin in the other panels of Fig. 3.
* We have updated the Figure accordingly.

4. It would be beneficial to readers to briefly describe what cell type the transgenic reporters label when mentioned in the Results section to help readers unfamiliar with zebrafish.
* We have updated the text to read:
`We further analysed heart morphology using live lightsheet microscopy of *Tg(myl7:LifeActGFP);Tg(fli1a:AC-TagRFP)* double transgenic embryos, allowing visualisation of myocardium (green) and endocardium (magenta) respectively. Analysis of heart morphology between 55hpf and 120hpf revealed that *llgl1* mutants continue to exhibit defects in heart morphogenesis (Fig S2I-N).'

Reviewer #2

Major comments
- The authors note an interesting observation with apical and basal laminin deposition dynamics surrounding cardiomyocytes, and that Llg1 has a role in apical Laminin deposition (however, highly variable at 80 hpf as Figure 3M shows). They carry out a very nice study in which they overexpress

© 2024. Published by The Company of Biologists under the terms of the Creative Commons Attribution License (https://creativecommons.org/licenses/by/4.0/).

Llgl1 tagged with mCherry in the myocardium and show that there is no rescue of the extruding cardiomyocyte defect or Laminin deposition. However, there is still a possibility that the tagged Llgl1 in the transgene Tg(myl7:Llg1-mCherry)sh679 might not be functional due to improper protein folding or interference by the mCherry tag. The authors should supplement their approach with a transplantation experiment to generate mosaic llgl1 mutant animals and assess whether llgl1 mutant cardiomyocytes extrude at a higher rate than the control. This would provide definitive evidence that Llg1l acts in a cell non-autonomous manner.
* We performed transplantation experiments to assess whether loss of *llgl1* specifically in CMs drives apical CM extrusion, transplanting wild type, het or *llgl1* mutant donor cells into wild type, het or *llgl1* mutant hosts. We generated 58 embryos with cells transplanted into the myocardium (new Table S1), however we only identified 4 embryos in which any transplanted cells were extruding (Fig 8), including 12 embryos with *llgl1* mutant cells in wild type/heterozygous hearts, and 9 embryos with wild type/heterozygous cells in *llgl1* mutant hearts. In that former group, if the loss of *llgl1* specifically in CMs was driving cell extrusion, we would expect a larger number of extruding transplanted cells, or more embryos with cell extrusion. To the contrary, we found that transplantation of *llgl1* mutant cells into wild type or heterozygous hosts did not result in large numbers of extruding mutant cells, or disproportionate numbers of embryos with extruding transplanted cells (Fig 8, Table S1). Similarly, wild type or het cells transplanted into *llgl1* mutants were still occasionally extruded. Together this suggests that specifically myocardial loss of *llgl1* does not result in CM extrusion, and supports our other data that epicardial defects in *llgl1* mutants cause aberrant apical CM extrusion.
- The data in this manuscript appears to point that Llgl1 regulates Laminin deposition mainly in epicardial cells to regulate their dissemination/migration across the ventricular myocardial surface. It would be important to test this cell-autonomous function with the transplant experiment (above point) and examine whether llgl1 mutant epicardial cells fail to migrate and deposit Laminin. It might be possible to perform a rescue experiment through overexpression of Llgl1 in epicardial cells (if possible, there is a tcf21:Gal4 line available).
* We performed the previously-described transplantation experiments to address the cell autonomy of *llgl1* in epicardial development as well as cardiomyocyte extrusion. We found that donor *llgl1* mutant cells were significantly less likely to form flattened epicardial cells and form epicardial cells on the outer curvature of the ventricle than wild type donor cells, supporting our hypothesis that *llgl1* drives timely epicardial development (Fig 5). Due to the technically challenging nature of transplantations and numbers required for robust analysis we did not directly assess Laminin deposition in transplanted embryos. We live-imaged the embryos to be able to assess both transplanted epicardial cells and/or myocardial cells (in line with our previous quantifications of CM extrusion). However we have also included data demonstrating that in *wt1a* morphants *lamb1a* and *lamc1* mRNA expression is lost in epicardial cells (Fig S4). Together our data now shows that 1) epicardial emergence and ventricular colonisation is delayed in *llgl1* mutants; 2) *llgl1* is required in epicardial cells for timely colonisation; 3) laminin deposition is delayed in *llgl1* mutants; 4) laminin subunit mRNA is expressed in epicardial cells; 5) loss of epicardial cells results in loss of laminin mRNA expression and protein deposition. Together we feel this supports our conclusions that *llgl1* promotes apical ventricular laminin deposition through timely epicardial colonisation.

- In the Discussion, the authors propose that Llgl1 acts in two ways: Laminin deposition in epicardial cells that suppress cell extrusion and polarity regulation in cardiomyocytes to promote trabeculation. It would be important to test the second hypothesis on trabeculation and polarity regulation by using the myocardial-specific overexpression/rescue of Llgl1 in llgl1 mutants, and then quantifying the trabeculating cardiomyocytes and analyze Crb2a localization. This experiment can distinguish whether this trabeculation phenotype is rescued independently of the apical Laminin deposition that has been included in Figure S5.
* To address this we analysed ventricular wall organisation in more details in wild type, heterozygote and *llgl1* mutant embryos either positive or negative for the *Tg(myl7:llgl1-mCherry)* transgene. However, we found upon analysing wild type embryos with the transgene that expression of *llgl1* under the *myl7* promoter was resulting in disorganisation of the ventricular wall, particularly onset of trabeculation (characterised by more trabecular cells). We therefore decided not to pursue the use of this model further, and have also removed from the manuscript the experiments and conclusions based upon the failure to rescue the *llgl1* extruding cell phenotype, since our wild type analysis suggests the transgene is not rescuing, but more likely also inducing overexpression effects in the mutant. This does also support our hypothesis that levels of

© 2024. Published by The Company of Biologists under the terms of the Creative Commons Attribution License (https://creativecommons.org/licenses/by/4.0/).

myocardial *llgl1* regulates ventricular wall organisation and trabeculation. We have decided to include this overexpression information into Figure S8 and describe our findings in the text since this overexpression phenotype could have disease implications (see extra sentence in the final paragraph of the discussion). We instead performed transplantation experiments to allow us to explore the cell autonomy of *llgl1* in cell extrusion (see responses elsewhere in this rebuttal for details). We did not use transplantation experiments to address the role of *llgl1* in Crumbs relocalisation, since this experiment would be extremely difficult to power appropriately given the low success rate of cell transplantation into the myocardium. However we did use it to solidify our epicardial data, since that is the larger narrative in our work.

- The potential mis-localization of Crb2a in the llgl1 mutants is interesting, but this effect appears to be quite mild, and as the authors note, resolve by 80 hpf. Considering the role of Lgl in Drosophila in shifting Crb complex localization during early epithelial morphogenesis, it would be worth performing the analysis at earlier timepoints (between 55 and 72 hpf) to determine whether Llgl1 is indeed important for the progressive apical relocalization of Crb2a.

* We have now expanded our analysis of Crb2a to assess whether localisation of Crb2a is disrupted at 55hpf in *llgl1* mutants. We find that in both wild type and *llgl1* mutant embryos at 55hpf that Crb2a is upregulated at cell-cell junctions, and there is no difference in localisation between genotypes. This data supports our conclusion that llgl1 is required for subsequent timely relocalisation of Crb2a, and has been included in Figure 2.

OPTIONAL: It might be worth testing other antibodies that could mark the apical (particularly aPKC which is known to phosphorylate and regulate the Crb complex) and basolateral domains (Par1, Dlg) of the cardiomyocytes to definitively conclude that the epithelial integrity of the cells is affected.

Although there are no reports of working antibodies marking the basal domain in zebrafish, there is at least a Tg(myl7:MARCK3A-RFP) line published (Jimenez-Amilburu et al. (2016)) - which the authors can inject to examine the localization in mosaic hearts.

* We assessed epithelial polarity and integrity in *llgl1* mutant further through analysis of aPKC expression, which is localised to the apical domain of epithelial cells. We found apical enrichment of aPKC in wild type cardiomyocytes at 55hpf (in line with previously published data, Merks et al, Nat Comms 2018), which is unaffected in *llgl1* mutants. We also analysed aPKC localisation at 72hpf (when we see defects in Crb2a in *llgl1* mutants) in both wt and mutant embryos. At this time point aPKC is less strongly apically-enriched in either wild type embryos or *llgl1* mutants, however we do see generally lower levels of aPKC in the *llgl1* mutant compared to the wild type. This data is now included in Fig S4.

- Have the authors quantified the numbers of total cardiomyocytes in llgl1 mutants to correlate how many cells are lost as a consequence of extrusion? What is the physiological impact of this extrusion (ejection fraction, total cardiac volumes, sarcomere organization)?

* We have now provided a more complete description of the *llgl1* mutant. This includes quantification of cell number at 55hpf and 80hpf (no significant difference between wt and *llgl1* mutants, which is in line with the previously published *llgl1* mutants). We do not see a difference in cell number at 80hpf – the numbers of extruding cells can vary significantly per heart (see Figure 1), and we do not believe that if these cells are leaving the heart, it would be possible to pick up a significant change, particularly since we observe defects in trabeculation. This data is now included in Figure S2. We also quantified ventricular size, ventricular myocardial tissue volume, and lumen volume, which also exhibit no significant differences between wt and mutant at 55hpf and 80hpf – this data is included in Figure S2 together with the initial characterisation of the mutant cardiac phenotypes.

We also characterised in greater detail cardiac function in our *llgl1* mutants. We find no defects in heart rate, blood flow velocity (in the trunk vasculature), ventricular shortening or systolic fraction in *llgl1* mutants compared to wild type embryos at 55-80hpf. This data is now included in Figure S5.

- The lamb1a and lamc1 mutant phenotypes were nicely analyzed. However, there is basement membrane deposition on both the apical and basal sides of the cardiomyocytes. Therefore, it is unclear whether the cardiomyocyte extrusion is completely caused by loss of apical basement membrane, or whether the loss of basal basement membrane could compromise the myocardial tissue integrity. The authors should clarify this conclusion in the text.

* To address this we have analysed Laminin expression at 55hpf in *llgl1* mutants, to determine whether basal laminin is inappropriately lost in *llgl1* mutants at an earlier timepoint that 72hpf,

© 2024. Published by The Company of Biologists under the terms of the Creative Commons Attribution License (https://creativecommons.org/licenses/by/4.0/).

which could result in compromised tissue integrity. We find no defect in basal laminin in *llgl1* mutants at 55hpf (we have now split the *llgl1* laminin data into two figures – the new *llgl1* data is in new Figure 4, along with enlargements of the previous images). This, combined with our observation that basal laminin levels in wild type embryos are generally equivalent to those in *llgl1* mutants, and that wild type embryos do not exhibit large numbers of extruding cells, suggests that defects in basal laminin are not responsible for tissue integrity defects in *llgl1* mutants. We have included a short sentence highlighting this in the discussion.

Minor comments
- The authors note that Llgl1-mCherry in the Tg(myl7:Llg1-mCherry)sh679 line localizes to the basolateral domain of the cardiomyocytes, which is valuable confirmation that Llgl1 protein is spatially restricted. However, only 1 timepoint (55 hpf) is noted. It would be important to perform Llgl1 localization across different developmental timepoints (at least until 80 hpf) to examine the dynamics of this protein during trabeculation and apical extrusion, and potentially correlate it with Crb2a localization for a better understanding of the apicobasal machinery in cardiomyocytes.
* We have decided not to include extra timepoints in the paper, for the reason that we are no longer using this transgenic line to provide supportive evidence that *llgl1* in the myocardium is not required to prevent apical CM extrusion. This is related to our response to an earlier comment from the reviewer around using the *myl7:llgl1* transgenic to assess other phenotypes in the *llgl1* mutant. We have retained the original images in the paper to demonstrate correct localisation of the protein (at 55hpf, the most relevant timepoint since trabeculation begins at around 60hpf), but this is now instead accompanied by quantifications demonstrating the impact of the transgene on trabeculation in wild type embryos, as we feel these are important data (and that the extra timepoints would now not add to the study). This data is now in Figure S8.
- The phenotypes of llgl1 mutants described here differ compared to the previous study by Flinn et al. (2020). In particular, whereas the mutants generated in this study have only mild pericardial edema and are adult viable, approximately one third of llgl1mw3 (Flinn et al. (2020)) died at 6 dpf. Is this caused by the different natures of the mutations in the llgl1 gene? Is there a possibility that the llgl1sh598 is a hypomorphic allele since the targeted deletion is in a more downstream sequence (in exon 2) compared to the llgl1mw3 (deletion in exon 1) allele?
* We thank the reviewer for noticing these subtle differences between the two *llgl1* mutants. Indeed, while we occasionally see *llgl1*^{sh598} mutants with the severe phenotype described by Flinn et al, this is a small minority which we did not quantify. Our mutation is indeed slightly further downstream than that described by Flinn et al, however we believe that this will have a negligible effect on Llgl1 function. Our *llgl1*^{sh589} mutation results in truncation shortly into the WD40 domain, and importantly completely lacks the Lgl-like domain, which is responsible for the specific function of Llgl1 likely through its ability to interact with SNAREs to regulate cargo delivery to membranes (Gangar et al, Current Biology 2005).
Interestingly, Flinn et al report no increased phenotypic severity in their maternal-zygotic *llgl1* mutants when compared to zygotic mutants. Conversely, we often observed very severe phenotypes in *MZ llgl1*^{sh589} mutants, including failure of embryos during blastula stages, apparently through poor blastula integrity. We did not include this information in the manuscript. However, we argue that together these differences between the two alleles may not be due to hypomorphism of our *llgl1*^{sh589} allele, but rather differences in genetic background that may amplify specific phenotypes. We have included the following text in the discussion:
'Llg1 has been shown previously to be important for heart morphogenesis (Flinn et al. 2020), and while similar defects in heart morphology are apparent between the *llgl1* mutant alleles, we also observe discrepancies. Most *llgl1*^{sh598} mutants are adult viable, representing a milder phenotype than the *llgl1*^{mw3} allele where a third of mutants do not survive to adulthood, however we found that some adult *llgl1*^{sh598} mutants respond poorly to anaesthesia, suggesting cardiovascular deficiencies. Conversely, we observed severe maternal effects of the *llgl1*^{sh598} mutation, with maternal-zygotic *llgl1*^{sh598} mutants exhibiting variable, but often severe, defects in blastula integrity (data not shown), which are not reported in *llgl1*^{mw3} mutants. These discrepancies may result from differences in genetic background that amplify specific phenotypes.'

Suggested experiment: qPCR of regions downstream of the deletion to make sure that the transcript is absent/reduced in the llgl1sh598 mutants. Alternatively, immunostaining or Western blot would be an even better option to ensure there is no Llgl1 protein production - there is an anti-Llgl1 antibody available that works for Western blots in zebrafish (Clark et al. (2012)).

© 2024. Published by The Company of Biologists under the terms of the Creative Commons Attribution License (https://creativecommons.org/licenses/by/4.0/).

* We have performed qPCR analysis of *llgl1* levels in sibling and *llgl1* mutant embryos, using two different primer sets that are downstream of our mutation. We see no significant downregulation of *llgl1* levels (this data is now included in Fig S1), however we do not interpret this as evidence that the mutation would result in a non-functional protein. Indeed, lack of NMD (nonsense mediated decay) may allow a better analysis of loss of *llgl1* function, since NMD of aberrant transcripts (including those containing PTCs) has been associated with activation of genetic compensation (El-Brolosy et al, 2019). The similarity in heart morphology phenotypes observed in $llgl1^{sh598}$ mutants to those published in Flinn et al, 2020, suggests that both mutants are loss-of-function.

- Closeups needed for Figure 3I-L' - difficult to assess mis-localization or differences in Laminin staining. Contrary to the quantification or conclusion, the Laminin staining appears stronger in llgl1 mutants compared to wild types in Figure 3I' and J'.

* We have now included magnifications in this figure (Now figure 4A' and B').

- OPTIONAL: Gentile et al. (2021) found that reducing heartbeat led to decreased cardiomyocyte extrusion in snai1b mutants. The authors could look into the contribution of mechanical pressure through contraction in the apical cardiomyocyte extrusion, and test whether reducing contraction (tnnt2 morpholino, chemical treatments) partly rescues the llgl1 mutant phenotypes.

* We believe there is a complicated relationship between cardiac function and myocardial wall integrity. The paper referred to by the reviewer indeed finds that reduction in heartbeat leads to decreased CM extrusion upon loss of the EMT-factor Snai1b. Previous studies have also found that endothelial flow-responsive genes *klf2a/b* are required to maintain myocardial ventricular wall integrity at later stages in a contractility-dependent manner (Rasouli et al, 2018). However, contractility is also required early for pro-epicardial emergence, but plays a lesser role in expansion of the epicardial layer on the myocardial surface (Peralta, 2013). Unpicking the relationship between the forces induced by mechanical contraction of the ventricular wall, contractility-based induction of e..g *klf2* expression, and the impact of contractile forces on proepicardial development or epicardial expansion will be complex. We therefore think the proposed experiment will be difficult to interpret whatever the outcome, and argue that dissecting this relationship is beyond the scope of revisions for this paper.

Reviewer #3
Major Comments:

(1) Major - the authors describe that llgl1 mutants exhibit transient cardiac edema at 3 dpf, which is resolved by 5 dpf, and claim that the mutants are viable. This statement needs to be better supported - What is the proportion of mutants that survive to adulthood? The embryonic phenotypes are pretty variable - are the mutants that survive the ones with a less severe phenotype? Is there a gross defect in the adult heart of these animals?

* We had some previous data from analysis of *llgl1* adults that we have now included in the manuscript with the following text:

'Despite the fact that *llgl1* mutants have morphological heart defects, they are adult viable. We observed no overt morphological abnormalities in adult *llgl1* mutants (Fig S3A-B), and found adult mutants at approximately Mendelian ratios in a colony grown from *llgl1* heterozygous incross embryos (wild type n=8, *llgl1+/-* n=11, *llgl1-/-* n=6). Since *llgl1* mutants exhibit variable heart morphology at 55hpf, we performed lightsheet imaging of *llgl1* mutant embryos at 120hpf, separated larvae into mild and severe cardiac phenotypes, and raised each category to adulthood. Adults raised from *llgl1* mutants with either mild or severe larval heart phenotypes had comparable morphology and behaviour. Swim tunnel analysis of *llgl1* mutants exercise tolerance revealed no deficits compared to wild type siblings, suggesting cardiovascular performance was not compromised (Fig S3C), and dissected *llgl1* mutant adult hearts appeared grossly normal. However, *llgl1* mutant fish responded poorly to anaesthesia, including gill bleeding (n=3/4), delayed recovery (n=3/4), and death (n=2/4), phenotypes which were never observed in wild type or heterozygous animals (n=6).'

(2) Major - Many of the phenotypes described here -most importantly, the defects on epicardial development- could result from hemodynamic defects in llgl1 mutants. The authors claim that function is unaffected in these animals, but this has only been addressed by measuring heartbeat. The observation that the cardiac function in these animals is normal would conflict with a previous description (PMID: 32843528) that demonstrates that llgl1 mutant animals show significant hemodynamic defects, which would cause epicardial defects. Thus, this aspect of the work needs to be better addressed.

© 2024. Published by The Company of Biologists under the terms of the Creative Commons Attribution License (https://creativecommons.org/licenses/by/4.0/).

* The previous study referenced describes defects in cardiac function in *llgl1* mutants at adult stages, and regurgitant blood flow in embryos at 72hpf (which could be the result of a dysmorphic atrioventricular canal). While they don't describe any contractile cardiac defects (and focus on valve defects) in *llgl1* mutant embryos, they do describe altered blood flow in *llgl1* morphant embryos which have a more profound phenotype than the mutant.
We therefore characterised in greater detail cardiac function in our *llgl1* mutants. We find no defects in heart rate, blood flow velocity (in the trunk vasculature), ventricular shortening or systolic fraction in *llgl1* mutants compared to wild type embryos at 55-80hpf. This data is now included in Figure S5. However, in line with our description above, we did observe in a small number of adult *llgl1* mutants a sensitivity to anaesthesia which is indicative of cardiovascular defects, in line with the previously published *llgl1* mutant.
The discrepancies between the two mutants are the result of partially overlapping analysis – we don't observe specific cardiac contraction defects in our *llgl1* mutant embryos, however in the previous study cardiac function was not directly assessed in llgl1 mutant embryos. The *llgl1* morphants described in the previous study represent the most severe phenotypes that we observe in our mutants, and there is little direct comparison in that study in terms of morphology or function of the morphants and mutants, thus comparison of blood flow velocity to our own mutant is not necessarily informative.
In the context of our own study, our data indicates that ventricular wall and epicardial defects in our *llgl1* mutants are not the result of abnormal cardiac function.

(3) The phenotypes related to forming multiple layers in the heart (Fig. 1) could be more convincing. In some figures, the authors use a reporter that labels the myocardial cell membrane, but in Figure 1 this is not used. Showing a myocardial membrane marker (for example, the antibody Alcama, Zn-8) would significantly strengthen this observation.
* We have now included example confocal slices through wild type and *llgl1* mutant hearts at 76hpf that demonstrate multilayering in the ventricle, including a nuclear transgene and DMGRASP staining to highlight membranes. This is included in Figure S2.

(4) The analysis of Crumbs redistribution (Fig. 2) is quite interesting. Still, given that the authors have a transgenic model to rescue llgl1 expression in cardiomyocytes, they could move from correlative evidence to experimental demonstration of the role of llgl1 in Crumbs localization.
* To address this (and comments from other reviewers suggesting using the *myl7:llgl1-mCherry* transgene to unpick the relative roles of *llgl1* in epicardial vs myocardial processes) we analysed ventricular wall organisation in more details in wild type, heterozygote and *llgl1* mutant embryos either positive or negative for the transgene. However, we found upon analysing wild type embryos with the transgene that expression of *llgl1* under the *myl7* promoter was resulting in disorganisation of the ventricular wall, particularly onset of trabeculation (characterised by more trabecular cells). We therefore decided not to pursue the use of this model further, and have also removed from the manuscript the experiments and conclusions based upon the failure to rescue the *llgl1* extruding cell phenotype, since our wild type analysis suggests the transgene is not rescuing, but more likely also inducing overexpression effects in the mutant. We have decided to include this overexpression information in Fig S8 and described our findings in the text. We instead performed transplantation experiments to allow us to explore the cell autonomy of *llgl1* in cell extrusion (see responses elsewhere in this rebuttal). We did not use transplantation experiments to address the role of *llgl1* in Crumbs relocalisation, since this experiment would be extremely difficult to power appropriately given the low rate of cell transplantation into the myocardium.

(5) (Optional) There is laminin in the luminal side of the heart before there is any epicardial invasion. What is the source of this laminin? The techniques the authors have used (i.e., chromogenic ISH) are fine, but a more detailed analysis using fluorescent ISH (i.e., RNAScope) would be much more definitive.
* This is related to our response to Reviewer 1 (above) – where we have included the following text included in manuscript: `Conversely, *wt1a* morphants retain deposition of laminin at the basal CM surface, likely from earlier expression and deposition of laminin by either myocardial or endocardial cells (Derrick et al. 2021; Nahia et al. 2023), which is unaffected by later epicardial development.` We hope this clarifies our proposed origins for the earlier laminin deposition.

(6) How llgl1 relates to epicardial biology is left entirely unexplored in this work. Do

© 2024. Published by The Company of Biologists under the terms of the Creative Commons Attribution License (https://creativecommons.org/licenses/by/4.0/).

proepicardial cells show any defect in cell polarization related to llgl1 absence?
* We agree with the reviewer that we do not delve into the mechanisms underlying regulation of epicardial development by *llgl1*, and that this is an interesting question. Our scope for this manuscript was to understand the mechanisms by which *llgl1* regulates integrity of the ventricular wall, and feel that uncovering the molecular mechanisms by which *llgl1* regulates timely epicardial emergence is a larger question that would require substantial investigation and investment beyond the scope of a revision. We think these are important questions that be better answered in depth in a separate manuscript.

Second decision letter

MS ID#: DEVELOP/2023/202482

MS TITLE: Llgl1 mediates timely epicardial emergence and establishment of an apical laminin sheath around the trabeculating cardiac ventricle.

AUTHORS: Eric JG Pollitt, Juliana Sanchez-Posada, Corinna M Snashall, Christopher J Derrick, and Emily Noel

Thank you for sending your manuscript to Journal of Cell Science through Review Commons.

I am happy to tell you that your manuscript has been accepted for publication in Development, pending our standard ethics checks.

Reviewer 1

Advance summary and potential significance to field

Pollitt et al. investigated the role of Llgl1 in maturation of the ventricular wall in zebrafish. They examined zebrafish heart morphology with microscopy analysis of fluorescent reporter lines crossed with their CRISPR/Cas9 llgl1 line and observed apically extruding CMs in llgl1 mutant embryos, implicating a role for llgl1 in ventricular wall integrity. Further, they examined apicobasal polarity in the ventricular wall through quantification of Crb2a distribution at the apical membrane surface, with enhanced Crb2a retention at CM junctions in llgl1 mutant embryos in comparison to WT at 72 hpf but similar levels at 80 hpf. Further analyses indicated a requirement for llgl1 for the temporal establishment of the apical laminin sheath, llgl1 is required for the timely dissemination of epicardial cells which deposit laminin to maintain the integrity of the ventricular wall. This work is written well and provides new information on heart development in zebrafish, and provides additional information on the role of the epicardium in supporting the integrity of the ventricular wall during trabeculation.

Comments for the author

Minor:
In Figure 8, panels N and O: It would be beneficial to include values indicating the significance, as p-values in figure legend indicate significance. Suggest adding stars to indicate significance, as in other figures.

Reviewer 2

Advance summary and potential significance to field

This is a revision from a previous manuscript submitted to Review Commons.

© 2024. Published by The Company of Biologists under the terms of the Creative Commons Attribution License (https://creativecommons.org/licenses/by/4.0/).

Comments for the author

Comments relevant to Reviewer 3 -
This is a reviewed manuscript that includes some advances compared to a previous version that I reviewed for Review Commons. The authors have been only modestly responsive to my comments. In taking a look at my comments and that of others reviewers, I would still advise to add data regarding the phenotype in adults.
This point was also voiced by reviewer 1, and it has been addressed only partially. Given that the authors do have the animals at hand, showing some histology of the adult hearts seems a very reasonable request.

Reviewer 3

Advance summary and potential significance to field

This summary was taken from my first assessment of the pre-revised manuscript:

The manuscript by Pollitt et al. explores the functions of llgl1, which encodes a critical component of the basolateral domain complex, during cardiac development in zebrafish. The authors observed that llgl1 mutants exhibited compromised myocardial tissue integrity with significantly higher numbers of apically extruding cardiomyocytes. Llgl1 appears to primarily function during epicardial cell spreading on the myocardial tissue, as myocardial-specific overexpression of llgl1 did not rescue llgl1 mutant phenotypes. llgl1 mutants exhibited impaired epicardial coverage and subsequently Laminin deposition on the apical side of the cardiomyocytes. Functional linkage between Laminin/the basement membrane was identified, as extruding cardiomyocytes were also observed in mutants of two core laminin genes, lamb1a and lamc1. The epicardial defects were transmitted to myocardial tissue defects, marked by mis-localization of the apical polarity protein Crumbs2a during early heart development. Overall, the authors provide a nice study that strengthens the role of apicobasal factors in myocardial tissue morphogenesis and that sheds light on the role of epicardial-derived basement membrane in maintaining myocardial tissue integrity.

Comments for the author

I thank the authors for thoroughly addressing my most major comments and experimental concerns. Particularly, the authors have done a great job in the transplant experiments to address the cell-specific requirements of llgl1, using the aPKC antibody to provide more evidence of the polarity defects seen in llgl1 mutants, and adding more quantitative analyses to assess the physiological effects of cell extrusion in llgl1 mutants. I have no more major concerns and comments, and think that the manuscript is suitable for publication in Development.

© 2024. Published by The Company of Biologists under the terms of the Creative Commons Attribution License (https://creativecommons.org/licenses/by/4.0/).
